# Do private health providers help achieve Universal Health Coverage? A scoping review of the evidence from low-income countries

**DOI:** 10.1093/heapol/czad075

**Published:** 2023-08-21

**Authors:** Laura Coveney, David Musoke, Giuliano Russo

**Affiliations:** The Wolfson Institute of Population Health, Queen Mary University of London, 58 Turner Street, London E1 2AB, United Kingdom; School of Public Health, Makerere University, New Mulago Hill Road, Mulango, Kampala, Uganda; The Wolfson Institute of Population Health, Queen Mary University of London, 58 Turner Street, London E1 2AB, United Kingdom

**Keywords:** Universal Health Coverage, health systems in LICs, private sector, private health providers, informal health services, primary care in LICs

## Abstract

Universal Health Coverage (UHC) is the dominant paradigm in health systems research, positing that everyone should have access to a range of affordable health services. Although private providers are an integral part of world health systems, their contribution to achieving UHC is unclear, particularly in low-income countries (LICs). We scoped the literature to map out the evidence on private providers’ contribution to UHC progress in LICs. Literature searches of PubMed, Scopus and Web of Science were conducted in 2022. A total of 1049 documents published between 2002 and 2022 were screened for eligibility using predefined inclusion criteria, focusing on formal as well as informal private health sectors in 27 LICs. Primary qualitative, quantitative and mixed-methods evidence was included, as well as original analysis of secondary data. The Joanna Briggs Institute’s critical appraisal tool was used to assess the quality of the studies. Relevant evidence was extracted and analysed using an adapted UHC framework. We identified 34 papers documenting how most basic health care services are already provided through the private sector in countries such as Uganda, Afghanistan and Somalia. A substantial proportion of primary care, mother, child and malaria services are available through non-public providers across all 27 LICs. Evidence exists that while formal private providers mostly operate in well-served urban settings, informal and not-for-profit ones cater for underserved rural and urban areas. Nonetheless, there is evidence that the quality of the services by informal providers is suboptimal. A few studies suggested that the private sector fails to advance financial protection against ill-health, as costs are higher than in public facilities and services are paid out of pocket. We conclude that despite their shortcomings, working with informal private providers to increase quality and financing of their services may be key to realizing UHC in LICs.

Key messages

- We used a UHC framework to scope the evidence of the contribution of private providers to advancing access to health services in LICs.
- We found evidence that most primary care, mother, child and malaria services are already made available by non-public providers across all LICs.
- Formal private providers mostly operate in well-served urban settings, while informal and not-for-profit ones cater for the lower end of the health market.
- Informal providers often provide services of suboptimal quality and fail to advance financial protection against ill health.
- Improving the quality and financing of informal providers may be key to expand UHC in LICs.

## Introduction and background

It is estimated that at least half the world’s population do not have access to basic healthcare, and nearly 100 million people are pushed every year into poverty paying for it (World Bank Group, 2017). Gaps in health provision are particularly stark in low-income countries (LICs), with most of those facing impoverishing health expenses based in low- and lower-middle income countries (LMICs) (World Bank Group, 2017).

Universal Health Coverage (UHC) has been defined as the global aim to ensure everyone receives the health services they need without suffering financial hardship (World Health Organisation, 2021; The Global Health Observatory, 2023), and has been conceptualized as a ‘cube’ comprising the three key dimensions of population coverage, service coverage and financial risk protection (World Health Organisation, 2010). Sustainable Development Goals 3.8 mentions achieving UHC for all by 2030, covering financial protection, access to quality healthcare services and access to safe, effective and affordable medicines and vaccines (World Health Organisation, 2021). Vast evidence (Torres-Rueda *et al.*, 2021; Rahman *et al.*, 2022) has been produced in the last decade showing that without financial protection, unexpected, catastrophic health expenditures can only paid through out-of-pocket payments (OOPP), often pushing the poor below the poverty line.

Most health systems around the world have a combination of public sector health services provided by the government and those that are privately owned or controlled (Klinton, 2020). The role of the private health sector within a country is defined according to its share of total health spending, its share of healthcare activity and the extent to which those seeking healthcare rely on OOPP (Mackintosh *et al.*, 2016). While it is known that the public and private health sectors interact, shape each other and the overall performance of the health system (Morgan *et al.*, 2016), their respective roles in achieving UHC are the subject of ongoing debate (Berendes *et al.*, 2011; Basu *et al.*, 2012; Horton and Clark, 2016).

Unclear boundaries are part of the reason why it has been so difficult to pin down a UHC role for the private sector (Mackintosh *et al.*, 2016). Mcpake *et al*. (2016) categorize private providers by their size, profit objectives and quality of services provided; the latter two are central issues in the debate on private sector involvement in UHC (Horton and Clark, 2016). Private-for-profit (PFP) providers are motivated by commercial interests and financial gain (Morgan *et al.*, 2016), while private not-for-profits (PNFPs) usually have a philanthropic motive (Klinton, 2020). I too may adopt similar funding streams (Mcpake and Hanson, 2016), but the central role of profit drives much of the debate on whether PFPs can adequately serve the poor, and whether either types of providers can be contracted by governments to advance UHC goals (Forsberg *et al.*, 2011) (Patouillard *et al.*, 2007; Pisani *et al.*, 2019).

The informal sector in LICs is typically seen as a widespread and heterogenous group of providers that include traditional birth attendants and healers, unlicensed drug shops and market stalls (Mills *et al.*, 2002), who provide a substantial proportion of health care in LICs for the poor, enabling them to access drugs and services that would otherwise be unreachable (Bloom *et al.*, 2011). A review of informal sector use in developing countries found that it provided between 9% and 90% of health care for some populations and health conditions (Sudhinaraset *et al.*, 2013). Although quality is lacking in both the public and private sectors in LICs (Berendes *et al.*, 2011), medical standards can be found wanting at the lower end of the market, while patient experience can be worse in public sector facilities (Basu *et al.*, 2012).

Much of the existing literature focuses on evaluating the performance of the private health sector in LMICs (Basu *et al.*, 2012), and concentrates on aspects of quality, equity and efficiency (Morgan *et al.*, 2016). We set out to extend this debate by systematically reviewing the recently published literature, with a specific focus on the private sector in LICs, and using the UHC metrics to understand progress. We scope the qualitative, quantitative and mixed-methods evidence from LICs, with the aim of understanding whether and how private providers have helped advance progress in population health coverage, breadth of services and financial protection. In the first section, we first explain the conceptual approach of our review, and the methods used to identify, select and analyse the evidence. We then present our results organized by the three UHC domains and other relevant themes emerging from the review. We conclude with a discussion of the key findings and on the contribution of our work to the existing bodies of knowledge on private health providers and UHC in LICs.

## methods

The key questions we aimed to answer through this review were:

- Is there evidence that private providers helped advance the achievement of UHC goals in LICs?
- What specific dimensions of UHC may have been advanced in such countries with the help of private providers: population coverage, breadth of services, financial protection or other aspects?

**Figure 1.**
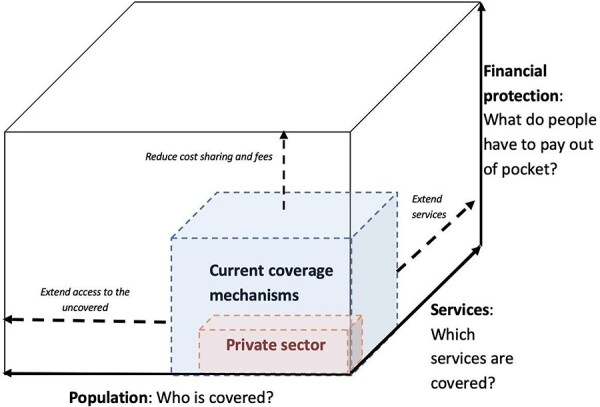
Private sector contribution to UHC

Source*: Authors’ own elaboration, adapted from Kutsin (2013) and the WHO UHC cube (**World Health Organisation, 2010*; *Kutzin, 2013*).

With a view to providing an answer to the above questions, we adapted the WHO’s UHC cube (World Health Organisation, 2010; Kutzin, 2013) to conceptualize the private sector’s contribution to UHC, placing a smaller cube within the larger one, to represent the role that the private sector plays in extending the three dimensions of coverage (Figure 1).

In this adapted model, the smaller private sector cube illustrates the ways the private providers can contribute to the traditional dimensions of overall breadth of healthcare services, population groups and financial risk protection within the mixed health system, borrowing the WHO definition of the private health sector (Klinton, 2020).

We carried out a scoping review of research on the extent to which private providers help extend the space of the cube across the three UHC domains of coverage of population, range of services and financial protection in LICs, as well as around emerging themes on the private sector’s role in progressing UHC. While traditional systematic reviews seek to answer narrow questions by assessing specific outcomes, scoping reviews identify knowledge gaps, scope a body of literature, clarify concepts or investigate research conduct (Arksey and O’malley, 2005). This suited our broad research question, providing a tool to map out diverse sources and types of evidence, identify the core concepts and provide an overview of the evidence around this topic (Arksey and O’malley, 2005). We used Arksey and Malley’s five-stage framework for scoping reviews (Arksey and O’malley, 2005) and the PRISMA extension for scoping reviews (PRISMA-ScR) checklist (Tricco *et al.*, 2018). Given the scoping nature of this review, there was no need to register the protocol beforehand.

### Selection criteria

To guide the selection process, we developed inclusion and exclusion criteria regarding types of providers and countries, dimensions of health coverage and type of evidence to be considered (Table 1). Our inclusion criterion for private sectors covered formal as well informal providers, as per the WHO recent operational definition (Klinton, 2020).

**Table 1.** Inclusion and exclusion criteria

	Inclusion criteria	Exclusion criteria
Private providers	Private-for-profit (service providers, pharmacies, insurance providers), not for-profit (NGOs, faith-based), informal providers (e.g. unlicensed drug outlets, hawkers and traditional healers)	Not specific to the private sector or providers—general evidence on the health system or public sector
UHC dimensions	At least one UHC dimension: Service coverage Population coverage Financial protection	Not specific to UHC or any of its dimensions—e.g. provider performance
Context	27 low-income countries from World Bank, 2022 list	Not specific to LICs or with disaggregated data on LICs From MICs and HICs.
Research type	Original research and analysis	Not a research paper. Commentary, opinion, editorials, letters
Peer review	Published in peer reviewed journals	Non-peer reviewed sources. Grey literature
Study designs/evidence considered	Quantitative, qualitative, mixed methods, case studies, health policy analysis, systematic reviews, original analysis of secondary data	Reproduction of secondary data or analysis, non-systematic reviews
Date	Published between 2002 and 2022	Published pre-2002
Language	Papers in English	Paper not in English
Access	Access to full text	Limited or no access

Using the Joanna Briggs Institute (JBI) ‘population, concept and context’ components, we defined our population of interest as the private health sector according to the broad WHO definition; the central concept was UHC underpinned by its three dimensions—population, service and financial coverage. The context for the review was the 27 LICs identified using the World Bank definition of countries with a gross national income (GNI) per capita of $1045 or less in 2020 (World Bank, 2022). We only included countries meeting this LIC classification at the time of the review, excluding former LICs and LMICs, on the assumption that by restricting the inclusion to countries classed as LIC at the time of writing, this would capture the very poorest where UHC is arguably most urgently needed.

We only included papers reporting findings from original research and analyses published in peer-reviewed journals. With a view to scoping the full breadth of evidence available on private providers in LICs, we included primary evidence obtained through quantitative, qualitative and mixed methods, systematic reviews and original analyses of secondary data. The timeframe for selected publications was set to 2002 to the time of the searches (June 2022) to cover the period of debate and research into UHC. Only papers written in English and for which the full text was available were included.

### Search strategy

We carried out literature searches in June 2022 across three electronic databases: PubMed, Scopus and Web of Science Core Collection. We developed search terms for each of the three population, concept and context (PCC) components (private sector, UHC and LICs) based on common terminology used in the existing literature, and ran searches for each ‘population, concepts and context’ component by linking individual terms using the ‘OR’ Boolean operator (e.g. universal health coverage OR universal access OR UHC). For the main searches in the PubMed database, this was followed by combining the three PCC component searches using the ‘AND’ Boolean operator:

(‘private sector’ OR ‘private provider’ OR ‘informal sector’ OR ‘traditional medicine’) AND (‘universal health coverage’ OR ‘universal access’ OR ‘UHC’) AND (‘LIC’ OR ‘low-income country’ OR ‘developing country’ OR ‘least developed country’)

Each of the 27 LICs was included in the LIC search component to expand the search results generated, as well as a range of other synonyms and related terms for private providers, such as ‘private hospital’ and ‘hawker’. The full search strategy with exact terms and number of results is shown in Supplementary Appendix 1.

### Selection, data extraction, analysis and reporting

We exported the search results from each database to Endnote and removed the duplicates. Study selection was carried out by two reviewers in three stages: title screening, abstract screening and full text screening using the predefined selection criteria, with reasons for exclusion recorded in an Excel spreadsheet. We extracted the data using a predefined charting template to improve consistency across a heterogeneous dataset, which included publication information (author, year, setting, evidence type), the private sub-sector(s) and provider type(s) studied and relevant data on each of the three UHC dimensions and quality of provision.

Although not specifically required for scoping reviews, we conducted a quality assessment in order to assess the overall quality of a very diverse evidence base. We used the JBI critical appraisal tools (JBI, 2022) to calculate scores for each paper by giving them a score against each criteria of either 1 point for a ‘yes’ or 0 for a ‘no’. Where criteria were not applicable to the study design, they were excluded from the calculation. Percentages were calculated for each article and reported in the results.

We reported the findings according to the PRISMA-ScR guidelines (Tricco *et al.*, 2018), using descriptive statistics to summarize the scope of the included evidence (study setting, evidence type, UHC themes addressed, quality score) and tabulating key findings for each article (private sub-sectors studied, UHC themes addressed), followed by a narrative summary of the evidence.

## Results

**Figure 2.**
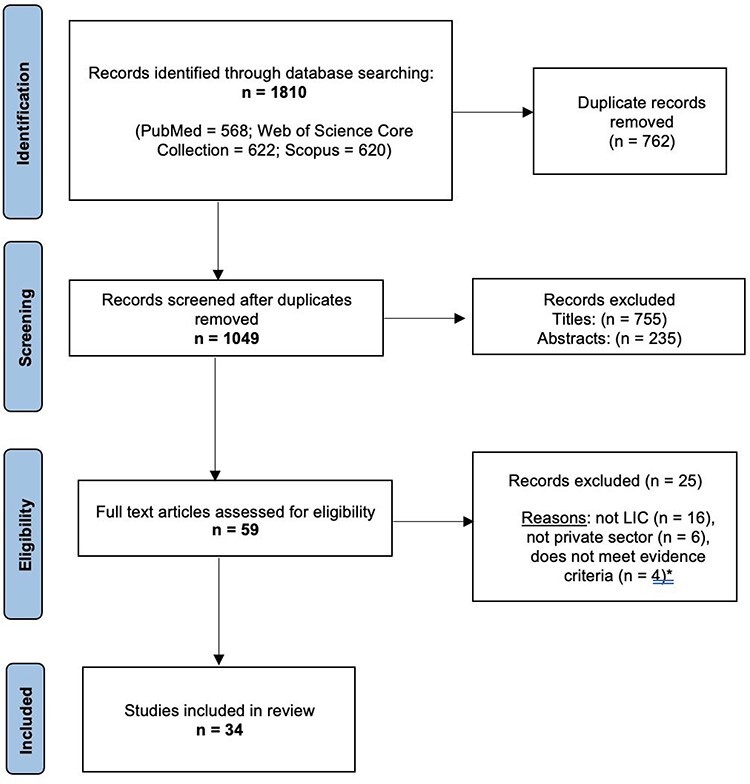
Selection process flow chart

The results of the literature search and selection process are shown in Figure 2. Of the 1810 records, 1049 unique papers were screened. From these, 59 studies were identified for full text screening, of which 34 met the eligibility criteria and were included in the review. In Supplementary Appendix 2 we list the studies excluded after full text screening, with reasons.

A total of 34 research papers, published between 2011 and 2021, met the review’s inclusion criteria. The key methodological characteristics and findings of each study are detailed in Supplementary Appendix 2, and Table 2 provides a summary of the included evidence. The studies provided direct evidence on 19 LICs, almost exclusively in sub-Saharan Africa. Two studies looked at LICs as a whole group, 12 were multi-country, seven were national, 11 sub-national and two focused on specific communities. For eight countries, we could not find studies providing direct and dedicated evidence on private providers and UHC (Burundi, Eritrea, Gambia, Guinea Bissau, Democratic People’s Republic of Korea, South Sudan, Syria and Yemen).

**Table 2.** Summary of evidence on private sector contribution to UHC—number of sources found in parenthesis

	Private sector			UHC dimension					
	Formal (29)		Informal (12)	Service coverage (29)		Population coverage (17)		Financial protection (20)	
Source/country	For-profit (27)	Not for-profit (14)	Informal (12)	Range (25)	Quality (14)	Socioeconomic group (8)	Geographic location (10)	Payment mechanisms (12)	Healthcare costs (10)
Abiiro *et al.*, 2014 (Malawi)	✓	✓		Qual	Qual		Qual	Qual	
Ameh *et al.*, 2021 (Multi-country)	✓				Qual				
Atake, 2020 (Togo)	✓				Quant			Quant	
Benova *et al.*, 2018 (Uganda)	✓	✓		Quant	Quant	Quant			
Beogo *et al.*, 2016a (Burkina Faso)	✓	✓	✓	Quant					Quant
Beogo *et al.*, 2016b (Burkina Faso)	✓	✓	✓					Quant	Quant
Campbell *et al.*, 2016 (Multi-country)	✓	✓	✓	Quant					
Chakraborty and Sprockett, 2018 (Multi-country)	✓	✓	✓	Quant			Quant		
Chirwa *et al.*, 2013 (Malawi)		✓		Mixed	Mixed	Mixed	Mixed	Mixed	
Doctor *et al.*, 2019 (Multi-country)	✓	✓		Quant		Quant			
Ewen *et al*., 2017 (Multi-country)	✓			Quant					Quant
Gele *et al.*, 2017 (Somalia)	✓			Qual	Qual				Qual
Grépin, 2016 (Multi-country)				Quant					
Hanson and Goodman, 2017 (Multi-country)	✓		✓	Quant					
Kasilo *et al.*, 2019 (Multi-country)			✓	Qual	Qual				
Khuluza *et al.*, 2017 (Malawi)	✓	✓	✓	Quant	Quant				Quant
Kiguli-Malwadde *et al.*, 2020 (Uganda)	✓			Quant			Quant		
Kim *et al.*, 2016 (Afghanistan)	✓			Quant			Quant		
Montagu and Chakraborty, 2021 (Multi-country)				Quant					
Nabyonga Orem *et al.*, 2011 (Uganda)	✓	✓	✓			Quant		Quant	
Namakula *et al.*, 2021 (Uganda)	✓				Mixed	Mixed		Mixed	
Ntambue *et al.*, 2018 (DRC)	✓	✓							Quant
Nyasulu *et al.*, 2019 (Malawi)	✓	✓	✓	Quant			Quant		
Orya *et al.*, 2017 (Sierra Leone, Somalia)			✓	Qual	Qual		Qual		
Palafox *et al.*, 2019 (Multi-country)	✓		✓				Quant	Quant	
Pettigrew and Mathauer, 2016 (Multi-country)	✓	✓						Quant	
Poyer *et al.*, 2015 (Multi-country)	✓			Quant					Quant
Sundari Ravindran and Fonn, 2011 (Multi-country)	✓			Mixed	Mixed		Mixed	Mixed	
Riley *et al.*, 2018 (Multi-country)	✓			Quant					Quant
Salim and Hamed, 2018 (Sudan)	✓			Qual	Qual	Qual		Qual	
Sisay *et al.*, 2021 (Ethiopia)	✓			Quant					Quant
Ssennyonjo *et al.*, 2018 (Uganda)		✓			Mixed	Mixed		Mixed	
Tan *et al.*, 2021 (Rwanda)			✓	Qual	Qual		Qual	Qual	Qual
Wang *et al.*, 2011 (Multi-country)	✓			Quant		Quant			

Source: Supplementary Appendix 3. Note: Qual: Qualitative evidence; Quant: Quantitative evidence; Mixed: Mixed methods evidence; DRC: Democratic Republic of Congo. Two papers had no disaggregated private subsector data (Grépin, 2016; Montagu and Chakraborty, 2021).

The research included 23 cross-sectional surveys, seven qualitative and four mixed methods studies. Most of the evidence was quantitative, based on cross-sectional household surveys, 12 of which drew on national Demographic and Health Survey (DHS) data. The study quality was good overall, with half of the papers scoring 90% or above using the JBI tool, 82% scoring over 80% and only one paper receiving a score of less than 60% (Supplementary Appendix 3).

The studies provided evidence on all three dimensions of UHC: 29 relating to service coverage, 16 on population coverage and 20 on financial protection (Table 2). They spanned six themes: range of services (25 papers) and quality (14); population coverage by socioeconomic group (9) and geographical areas (10); and financial protection including payment mechanisms (12) and health care costs (10).

We organized the narrative analysis firstly by summarizing the nature of the private sector in LICs, and secondly, describing the evidence under each of the three UHC dimensions, with quality of care as a subtheme within the service coverage dimension.

### The diverse private sector

The included studies covered a wide range of private provider types, illustrating the diverse nature of the private health sector in LICs (Supplementary Appendix 3). Table 3 summarizes the specific provider types covered in the research, organized according to the profit-quality categories set out in the private sector cube framework. Overall, two papers looked at the private sector, while 29 studies reported evidence on formal private providers, and 12 on informal ones, demonstrating a more developed evidence base on the formal private sector in LICs.

**Table 3.** Different types of private provider in LICs, as identified by the review’s papers

	For-profit	Not for-profit
Formal	Commercial health centres (Gele *et al.*, 2017, Ntambue *et al.*, 2018, Poyer *et al.*, 2015) Hospitals (Kim *et al.*, 2016) Clinics (Abiiro *et al.*, 2014, Beogo *et al.*, 2016a, Nabyonga Orem *et al.*, 2011, Namakula *et al.*, 2021, Palafox *et al.*, 2019, Riley *et al.*, 2018, Gele *et al.*, 2017) Pharmacies (Khuluza *et al.*, 2017, Abiiro and De Allegri, 2015, Palafox *et al.*, 2019, Poyer *et al.*, 2015, Riley *et al.*, 2018) Licenced drug stores (Khuluza *et al.*, 2017, Abiiro and De Allegri, 2015, Nyasulu *et al.*, 2019, Palafox *et al.*, 2019, Poyer *et al.*, 2015, Riley *et al.*, 2018) General retail outlets (Riley *et al.*, 2018) Laboratories and imaging centres (Namakula *et al.*, 2021) Private insurance providers (Atake, 2020, Namakula *et al.*, 2021, Pettigrew and Mathauer, 2016, Salim and Hamed, 2018) Social franchises (Sundari Ravindran and Fonn, 2011)	NGOs (Beogo *et al.*, 2016a, Ntambue *et al.*, 2018) Faith-based hospitals and clinics (Abiiro and De Allegri, 2015, Beogo *et al.*, 2016a, Chirwa *et al.*, 2013, Khuluza *et al.*, 2017, Ssennyonjo *et al.*, 2018)
Informal	Traditional healers and marabouts (Beogo *et al.*, 2016a, Beogo *et al.*, 2016b, Kasilo *et al.*, 2019, Abiiro and De Allegri, 2015, Tan *et al.*, 2021, Nabyonga Orem *et al.*, 2011) Traditional birth attendants (Orya *et al.*, 2017) Unlicensed drug stores (Campbell *et al.*, 2016, Nyasulu *et al.*, 2019) Mobile drug vendors (Hanson and Goodman, 2017) Market stalls and kiosks (Khuluza *et al.*, 2017, Palafox *et al.*, 2019)	Self-treatment (Beogo *et al.*, 2016b, Beogo *et al.*, 2016a)

Source: Supplementary. Appendix 3.

#### Types of formal private sector described in the literature

The formal private sector has been studied more extensively than the informal sector, with most studies relating specifically to the formal PFP sub-sector. Twenty-seven studies reported evidence on formal PFPs involved in delivering a wide range of different health services, mostly using cross-sectional survey data, demonstrating wide variation in size, complexity and quality within just the formal for-profit health sector. There was less variation among the NFP types discussed, which were mostly ‘health facilities’ run by NGOs or by faith-based organizations (Table 3).

#### Types of the informal private sector

There were fewer published studies on the informal health sector, with 12 papers that reported evidence on its role in malaria treatment, maternal and child health or UHC progress overall. All of these studies looked at informal providers that operate for profit, six researched traditional healers or birth attendants and five that looked at informal drug outlets. Two articles, both looking at household health expenditure in Burkina Faso, included self-treatment as a source of informal healthcare (Beogo *et al.*, 2016b); therefore, self-treatment has been categorized in this review as informal not-for-profit provision.

### Service coverage

The service coverage dimension of UHC was addressed in 29 of the included studies, (Supplementary Appendix 3). Of these, 25 looked at the range of private health services and products available, and 13 looked at their quality. Overall, there was evidence that the private sector provides a significant level of service coverage among LICs, with variable choice, availability and quality.

#### Range of services

Overall, we found in twenty studies that the private sector made a significant contribution to expanding the range of available services in LICs. A secondary analysis of DHS data showed that Uganda was the most privatized LIC, with 40% of all care delivered by private providers, including 21.4% of inpatient and 60% of outpatient care (Montagu and Chakraborty, 2021). A household survey from Afghanistan found that 13% of the patients interviewed used the private sector for outpatient care while less than 7% used the public service, although the reverse trend was true for inpatient care (Kim *et al.*, 2016). Gele *et al*. presented qualitative evidence from Mogadishu, Somalia, in which the PFP sector was widely perceived to be the main source of any health service, filling a gap left by the public sector, despite concerns over cost and quality (Gele *et al.*, 2017). The private sector was also found to be active in the provision of HIV-related services, although this varied across LICs, with between 8–24% of women and 14–24% of men reporting the PFP sector as their source of the most recent HIV testing, or for the NFP sector, between 0–7% for women and 0–16% for men (Wang *et al.*, 2011).

Five studies provided evidence that the private sector makes an important contribution to maternal health services, although this varied across countries and over time (Chirwa *et al.*, 2013; Abiiro and De Allegri, 2015; Grépin, 2016; Benova *et al.*, 2018; Doctor *et al.*, 2019). Between 1990 and 2013, a quarter of antenatal care (ANC) and 22% of institutional deliveries were provided in the formal private sector in LICs (Grépin, 2016), while there was wide variation between LICs over time, with private facility births soaring by 435% in Ethiopia from 5% between 2000 and 2016, while they dropped in the DRC from 20.5% to 15% (Doctor *et al.*, 2019).

Children’s healthcare was another highly privatized service reported in the literature, with five studies providing evidence of significant PFP and NFP involvement (Chirwa *et al.*, 2013; Abiiro and De Allegri, 2015; Grépin, 2016; Chakraborty and Sprockett, 2018; Nyasulu *et al.*, 2019). In another study, DHS data showed that between 10% and 42% of children’s diarrhoea and fever care was privately delivered in LICs, with PFP clinics and pharmacies providing about 25% of care in the DRC, 20% in Liberia and 10% in Mali, while informal providers accounted for 10% in the DRC, 20% in Liberia and 42% in Mali (Chakraborty and Sprockett, 2018).

A multi-country study using DHS data reported wide variation in the level of private sector delivery, finding that PPs provided between 2% of family planning services in Chad and 25% in Uganda (Campbell *et al.*, 2016). A similar study found that 30% of family planning services in Liberia were delivered in the PFP sector and around a quarter in Mali (Chakraborty and Sprockett, 2018). However, choice of contraceptives was found to be lower among PFPs in Ethiopia and the DRC, with less than 8% of private drug outlets in the DRC offering at least three contraceptive methods (Riley *et al.*, 2018).

Four papers presented mixed evidence on the extent of private providers extending malaria services, of which the majority were publicly provided with some private sector contribution to expanding product choice (Poyer *et al.*, 2015; Beogo *et al.*, 2016a; Hanson and Goodman, 2017; Khuluza *et al.*, 2017). In a series of retail surveys, most malaria testing in 2014/15 was found to be publicly provided in Madagascar (84.5%), Uganda (71.8%) and the DRC (74.3% in Katanga region and 51.9% in Kinshasa), although nearly half of private facilities that stocked antimalarials in Uganda offered rapid diagnostic testing (RDT), and 44% of them did in Madagascar (Hanson and Goodman, 2017).

Three studies provided evidence of the informal private sector complementing and expanding formal health provision (Orya *et al.*, 2017; Kasilo *et al.*, 2019; Tan *et al.*, 2021). Local traditional healers in rural Rwanda were perceived by the community to address a gap in health care provision, providing accessible treatments for minor health conditions and the culture-specific illness uburozi (poisoning), which formal clinics were not equipped to handle, and referred on to the Traditional Healers (Tan *et al.*, 2021). Traditional medicine was also found to play an important role in providing the health system with new, effective treatments for a range of health conditions in Burkina Faso, the CAR, Ethiopia, Malawi, Mali, Mozambique, Niger and Uganda (Kasilo *et al.*, 2019).

#### Quality of the services provided by private providers

Evidence of good quality care among private providers was reported in six studies (Chirwa *et al.*, 2013; Abiiro and De Allegri, 2015; Benova *et al.*, 2018; Salim and Hamed, 2018; Ssennyonjo *et al.*, 2018; Ameh *et al.*, 2021). In one, focus groups and interviews with community members in rural Malawi found that both PFP and the faith-based PNFP provided higher quality care than the public services, with better facilities and more time spent with health care workers (Abiiro *et al.*, 2014). A DHS data analysis from Uganda found no significant difference in patients’ perceptions of quality between public and private sector provision on ANC or deliveries (Benova *et al.*, 2018). However, Ameh reported that while formal PFP primary health services were with better quality with shorter waits than public provision, they were too expensive for a lot of people (Ameh *et al.*, 2021), while a study in Sudan reported that Private Health Insurance (PHI) offered access to higher quality private clinics, but policies were not affordable for many (Salim and Hamed, 2018).

Five articles presented contrary evidence, reporting that both technical and service quality were low in the formal private sector (Sundari Ravindran and Fonn, 2011; Gele *et al.*, 2017; Khuluza *et al.*, 2017; Atake, 2020; Namakula *et al.*, 2021). In Somalia, community members reported that quality was very low among PFPs in Mogadishu, especially for poorer patients, with low quality drugs being prescribed and providers not treating users with respect, which they blamed on unregulated profiteering and a lack of enforcement of guidance or standards by the government, eroding trust and preventing attendance when sick (Gele *et al.*, 2017). Quality issues were identified among PFP clinics participating in social franchises in Ethiopia, with problems in implementing training programmes, organizing monitoring visits and taking action against clinics when poor quality was found, due to difficulties in recruiting and retaining franchisees (Sundari Ravindran and Fonn, 2011).

The quality of informal sector provision was explored in four further studies, finding that informal providers were usually perceived as offering a good level of service quality due to their community-embedded position, although evidence on following clinical guidelines was not presented (Khuluza *et al.*, 2017; Orya *et al.*, 2017; Kasilo *et al.*, 2019; Tan *et al.*, 2021). A survey on 12 different antimalarial and antibiotic medicines was collected from 31 health facilities and drug outlets in southern Malawi, and found that illegal street vendors sold more substandard medicines than the public or NFP providers (Khuluza *et al.*, 2017).

Interviews with maternal health service users in rural Somalia and Sierra Leone showed that traditional birth attendants posed a risk to life for expectant mothers due to lack of technical skills, until they undertook training (Orya *et al.*, 2017). Similarly, in Rwanda, community members highlighted that traditional health providers offered more culturally appropriate care than formal Western health clinics, for example for treatment of *uburozi* (witchcraft) within the community (Tan *et al.*, 2021).

### Population coverage

Data from 17 studies on private sector contribution to population coverage suggested that the PFP sector tends to serve wealthier urban groups and offer ad hoc fee waivers for the poor, while the NFP and informal sectors are more likely to serve poorer, rural communities.

#### Coverage among specific socioeconomic groups

Private providers were found to primarily serve wealthier households in six of the included articles (Nabyonga Orem *et al.*, 2011; Wang *et al.*, 2011; Benova *et al.*, 2018; Salim and Hamed, 2018; Doctor *et al.*, 2019). An analysis of DHS data across SSA countries found a significant difference in private facility use by women from wealthy vs poor quintiles in the DRC (49% vs 5%), Ethiopia (8% vs 0.1%), Liberia (31% vs 3%), Mali (8% vs 1%), Niger (2% vs 0.3%), Rwanda (4% vs 0%) and Uganda (32% vs 8%) (Doctor *et al.*, 2019). Another multi-country study found that the use of private HIV testing was highest among women in the highest quintiles (73% as opposed to 34% in the lowest quintile), including in Ethiopia which had very high levels of disparity, with 99% of women and 93% of men who used private HIV testing coming from the wealthiest group (Wang *et al.*, 2011).

However, a mixed-methods study in Uganda reported widespread use of informal pro-poor approaches among PFPs, such as offering free or discounted care to the poor and charging wealthier clients more to compensate for lost profit, enabling the poor to access medicines, family planning and STI testing, although fee reduction was not offered for more complex interventions such as surgery or imaging (Namakula *et al.*, 2021).

NFPs were found to address socioeconomic gaps in cover in two studies (Chirwa *et al.*, 2013; Ssennyonjo *et al.*, 2018). A case study of the Uganda Catholic Medical Bureau provided evidence of increasing access to the poor through provision of free services; however, user fees was gradually reintroduced to cover rising costs, creating barriers to those unable to pay (Ssennyonjo *et al.*, 2018). Conversely, another case study, looking at the Christian Health Association of Malawi (CHAM), showed that, while NFP services had charged user fees that excluded the poorest, introduction of government Social Level Agreements helped to remove these barriers and increase population coverage (Chirwa *et al.*, 2013).

#### Geographical coverage

Evidence from five studies suggested that the PFP sector tends to be located in already well-served urban areas (Sundari Ravindran and Fonn, 2011; Kim *et al.*, 2016; Chakraborty and Sprockett, 2018; Nyasulu *et al.*, 2019; Kiguli-Malwadde *et al.*, 2020). In Afghanistan, women in urban areas were more likely to use private health facilities for ANC services (56%) compared with rural women (21%), which was a statistically significant difference (Kim *et al.*, 2016). In Uganda, an audit of imaging equipment found that the majority (75%) was privately owned, most of which was located in the central region where the capital city is, despite the population being dispersed across the country’s regions (Kiguli-Malwadde *et al.*, 2020). An analysis of social franchises of family planning services found that no new clinics were set up in areas not already served (Sundari Ravindran and Fonn, 2011).

There was limited evidence of the private sector extending geographical coverage of malaria services in Uganda (Palafox *et al.*, 2019). A mixed methods study in Malawi found that contracting faith-based NFPs in remote areas helped to reduce geographical barriers to accessing child and maternal health services (Chirwa *et al.*, 2013). Similarly, a qualitative study in rural Malawi found some limited PFP and NFP provision of essential primary health services in underserved rural parts, but they were often too expensive for users to access (Abiiro *et al.*, 2014).

Informal provision was found to play an important role in rural health coverage in Sierra Leone and Somaliland, where NGO-trained traditional birth attendants increased rural pregnant women’s access to institutional deliveries, although such figures may not be entirely considered as private sector (Orya *et al.*, 2017). Similarly, traditional healers in rural Rwanda provided local community members with easy to access care for minor illnesses and culture-specific sickness (Tan *et al.*, 2021).

### Financial risk protection

Twenty studies provided evidence on two key aspects for UHC financial risk protection in the private sector: the use of ‘protective’ payment mechanisms and the impact on cost of healthcare services. In Supplementary Appendix 3, our data extraction table showed that the use of OOPP was higher in the formal PFP sector, with greater pro-poor protection provided by NFPs and informal providers.

#### Payment mechanisms

There was strong evidence of high levels of OOPP use in the private sector, provided by nine studies (Nabyonga Orem *et al.*, 2011; Sundari Ravindran and Fonn, 2011; Abiiro and De Allegri, 2015; Beogo *et al.*, 2016b; Ssennyonjo *et al.*, 2018; Palafox *et al.*, 2019; Atake, 2020; Namakula *et al.*, 2021; Tan *et al.*, 2021). In urban Burkina Faso, a survey found significantly higher OOPPs among private providers, with patients who sought care from private facilities spending 48% more OOP than those in public facilities, which was 141% more than those who opted for self-treatment (Beogo *et al.*, 2016b).

Some PFPs informally provided financial protection on an ad hoc basis for those who could not afford to pay, waiving fees, providing loans or subsidizing costs by charging wealthier clients more (Sundari Ravindran and Fonn, 2011; Namakula *et al.*, 2021). Similarly, qualitative evidence on the informal sector showed traditional providers also offering flexible payment methods, such as loans or fee waivers (Tan *et al.*, 2021).

There is evidence that government funding reduced the use of OOPP by NFPs, until the level of financial support fell too low and fees needed to be reinstated (Chirwa *et al.*, 2013; Ssennyonjo *et al.*, 2018). In Togo, a household survey found that PHI offered policy holders lower monthly OOPP than Social Health Insurance holders. However, 36% of PHI holders did not seek medical care when sick, of which 43% said was due to OOPPs (Atake, 2020). Similarly, a study in Sudan found PHI policyholders had lower co-payments for drugs than SHI holders, but had to pay higher enrolment fees, which was less affordable for lower income households (Salim and Hamed, 2018).

#### Costs of health care

Eight studies looked at the costs of health care products and services and identified higher costs in the private sector, especially the formal for-profit sub-sector, whereas many essential services were offered for free in the public sector, where they were available (Poyer *et al.*, 2015; Beogo *et al.*, 2016a; 2016b; Gele *et al.*, 2017; Khuluza *et al.*, 2017; Ntambue *et al.*, 2018; Riley *et al.*, 2018; Sisay *et al.*, 2021).

A survey of contraceptive outlets in the DRC and Ethiopia found that, while most contraceptives were free from the public sector, private providers charged for all methods, with oral contraceptives costing on average between $0.33 and $0.88 in the DRC and between $0.15 and $0.36 in Ethiopia, and average charge for IUDs between $2.20 and $66.22 in the DRC and between $0.24 and $1.46 in Ethiopia (Riley *et al.*, 2018). Similarly, a survey in Malawi found that public facilities provided free malaria medicines, while 10/12 courses of treatment from both NFP and PFP providers cost more than the $1.25 average daily wage of the lowest-paid workers (Khuluza *et al.*, 2017). Another study from Ethiopia, reviewing prices of essential drugs, identified more unaffordable drugs in the private sector than the public, with 92% of medicines being unaffordable in private facilities, compared with 72% in the public sector, and with the top priced products costing between 63 and 186 days wages from private providers, compared with 40–71 days wages from public health providers (Sisay *et al.*, 2021).

A comparison of the charges for maternal services in the DRC found that private sector deliveries were more expensive than public, costing women $42 or $49 at PFP providers for an uncomplicated or complicated birth, respectively, and $47 or $57 at NFP providers, compared with $39 and $45 in the public sector (Ntambue *et al.*, 2018). However, they found the private sector to be more cost-effective, with an incremental cost effectiveness ratio of $56, against a threshold of $460, due to the higher availability of emergency interventions, and three times more women in private care than in the public sector receiving at least one (Ntambue *et al.*, 2018).

The informal sector appeared to represent the cheapest option (Beogo *et al.*, 2016a; 2016b; Tan *et al.*, 2021). In Burkina Faso, a survey found that informal treatment for malaria was the least expensive option, costing on average $5.20 compared with $17 in the public sector and $24.50 in the formal private sector (Beogo *et al.*, 2016a). In Rwanda, interview data indicated that traditional health providers charged between $2 and$5 for an initial consultation, and then accepted a follow-up fee or gift after the patient had recovered, and some provided care at no cost for the poor (Tan *et al.*, 2021).

## Discussion

Our work builds on previous reviews on this topic—such as (Morgan *et al.*, 2016)—and extends the evidence base on role and impact of private health providers in LICs in three meaningful ways. First, it considers exclusively the evidence on LICs, which are, arguably, a less diverse set of countries than the previously reviewed LMICs, with more similar private sectors. Secondly, it examines the published evidence following the formalized scoping review methodology. Thirdly, it explicitly uses the UHC dimensions and terminology to search the literature and organize the account of the modalities private providers contribute to expand health coverage.

This review identified 34 papers that met our inclusion criteria and reported evidence on the contribution of private health providers to achieving UHC in LICs. Evidence was found that most basic health care services in countries like Uganda, Afghanistan and Somalia are provided through the private sector. A substantial proportion of mother, child and malaria services are also made available through non-public providers across the 27 LICs, particularly malaria tests and first-line malaria treatments. Some evidence was found on private provision of traditional medicine integrated with standard health care in low-income settings. On the private sector’s capacity to extend population coverage, we found that, while formal private providers typically operate in already well-served urban settings, informal and not-for-profit ones cater for patients in underserved rural areas and generally at the lower end of the health market. Nonetheless, there is evidence that for many informal providers the quality of the products provided is suboptimal. A few studies suggested that the private sector broadly fails to advance financial protection against ill health in LICs, as private services appear to be available at higher costs than public ones and financed predominantly Out of Pocket.

Our findings need to be interpreted with a degree of caution. Because of the great diversity of private health sectors across LICs (Mackintosh *et al.*, 2016), it was not possible to arrive at firm conclusions on the role of specific providers for the advancement of UHC (see the next discussion point). Secondly, the current lack of definition on what represents progress towards UHC (Wagstaff *et al.*, 2016) made the task of identifying the evidence on the private sector’s impact inherently difficult. Many of the publications we reviewed did not consider the impact of illegal charges and health workers’ dual practice on public services (Leonard, 2005). As such phenomena reduce affordability and access to services for the poor, the boundaries between public and private sectors become blurred, calling into question traditional operational distinctions (Mcpake *et al.*, 2016). Finally, the interconnectedness of the effects of health service consumption (Endalamaw *et al.*, 2022) turned the attribution of the benefits to one of the three UHC domains a slightly artificial exercise. Our review focused exclusively on the published literature. While this is widely considered the gold standard of scientific research, we are aware that relevant evidence may have been missed, particularly about informal providers. Despite these limitations, a few useful reflections can be drawn from our review that might be used to expand the provision of healthcare services and improve the health status for low-income populations.

Although our inquiry focused on a sub-set of 27 potentially less diverse LICs, we found a greater than expected diversity of private providers. The private sector typologies developed by other scholars (Mcpake and Hanson, 2016) helped distinguish between the UHC contribution from formal, informal, for profit and not-for-profit providers. Within such categories, our evidence suggests that formal PFP providers in LICs somewhat manage to extend the breadth of services available within urban areas (Grépin, 2016), but not the geographical coverage. On the other hand, informal providers extend coverage across geographically hard-to-reach populations and lower socio-economic groups, but only for basic services, and with very uneven quality (Abiiro *et al.*, 2014). Somewhere in between, we found evidence that not-for-profit providers successfully extend population health coverage across the three dimensions of the cube (Ssennyonjo *et al.*, 2018), but as these are too few and far between, they are probably not in the position to make a significant difference to the national supply of services. Building on such findings, future studies will need to focus on each one of these types of private providers individually, and produce evidence applicable to countries with similar examples of private sector provision.

Our review found limited evidence on the quality of the services provided across the spectrum of private providers in LICs, with the notable exception of a systematic review that did not support the view that the private sector in LMICs is more medically effective and efficient than the public sector (Basu *et al.*, 2012). Although admittedly different criteria were often used to assess quality of services (technical quality of services, adherence to clinical guidelines or patients perceptions), there did appear to be systematic differences between products and services from formal private providers, and those available in the informal sector, which are typically of inferior contents (Kaur *et al.*, 2008; Kumah, 2022). On the other hand, Supplier-Induced Demand theory suggests that over-provision or prescription of higher-cost services is likely in the private sector (Tangcharoensathien *et al.*, 2019), which may further dent overall quality of services and universal coverage. As it is vital that health care services and products possess the minimum acceptable medical quality, it is somewhat surprising that this attribute has not been featured more prominently in the evolution of the UHC narrative. Because of the very diverse range of healthcare services and products found in low- and middle-income settings (Cox *et al.*, 2017), an argument could be made that quality and clinical effectiveness should be considered a core principle of universal coverage, and represent an additional dimension of the UHC cube in its own right. This would resonate with the strand of the literature pressing for quality of care to be enshrined in the Sustainable Development Goals at the heart of health systems worldwide, and not just considered as the purview of elites in higher-income countries (Kruk *et al.*, 2018).

Our work contributes to the ongoing debate on the role of the private sector in the provision of health services in low-income settings (Hanson *et al.*, 2008). On the one hand, the evidence uncovered on formal PFP providers suggests that, although they have a positive role in expanding choice of services in LICs, they might also be widening health inequalities by providing additional services to comparatively better served urban consumers (Harris *et al.*, 2011). On the other hand, we show that informal and NFP health providers occupy a space neglected by formal institutions, and provide services where there is little to no alternative. Rather than outright prohibition (Montagu and Goodman, 2016), regulatory efforts might be better directed at ensuring that these informal actors provide—for a profit—products of at least a minimum quality to hard-to-reach populations, and that they are sufficiently integrated into the health system to be able to refer cases that they cannot treat on to higher levels of care.

In future, greater evidence will be needed to assess the contribution of private providers to UHC. First, improving the metrics of UHC should be a priority, as the current UHC service coverage index (WHO, 2021) is not sufficiently refined to fully quantify progress, attributions and specific contributions. Secondly, rather than referring to the broad private sector, future studies should focus on specific formal or informal providers and services, with a view to identifying what works in improving progress to UHC, in what circumstances, and through what mechanisms. And finally, more evidence is needed on the quality of private providers, particularly for informal ones in rural areas, if these are to be used to sustain progress of coverage.

More to the point, our study seems to confirm the view that private providers can only be fully understood within the context of their respective mixed health systems (Mackintosh *et al.*, 2016). As public and private sectors interact, it is probably the forms and outcomes of such interactions that should be the subject of future research. Such view of the private sector would therefore beg for evidence for topics such as the trade-offs of health workers working concomitantly in public and private services, therefore implicitly subsidizing the services to the poor through the fees charged to the wealthy. By the same token, the effects of temporary aid-supported health services on the development and sustainability of local private providers should be examined, just like the competition for paying customers between public and private providers. Or the need for more convincing evidence on the common claim that private providers would help unburden public services.

Scholars have also suggested that private providers may be used as a delivery channel for essential publicly funded services, aligning the private sector to public health priorities, such as reducing health disparities and expanding access (Mcpake and Hanson, 2016). However, our review shows that such a solution does not appear to give sufficient consideration to the economic drive and opportunism at the base of most private enterprises (Russo and Mcpake, 2009). As an alternative, Montagu and Goodman posit that, while strong regulatory capacity is the long-term priority for LICs, encouraging quality improvement and enhanced coverage among private for-profits may be a more realistic goal in the near term (Montagu and Goodman, 2016). But rather than working with the formal private sector essentially focused on the most lucrative segments of the market, perhaps there may be more scope to engage with informal providers, and promise legalization of their activities in exchange for minimum quality standards or the provision of socially valuable health services.

Enabling informal providers to enter the formal market may also introduce greater competition, drive down prices while maintaining enough quality not to lose customers. Strengthening the system at the bottom may also increase acceptance of traditional health services and reduce the cultural barriers faced by formal public and private providers at the lower end of the market (Gilson, 2007) (Dyer *et al.*, 2016). To this respect, Morgan and colleagues argue that improving the performance of private providers requires interventions that target the health sector as a whole (Morgan *et al.*, 2016). If current informal private providers can be shaped to offer low-cost services of an acceptable quality, a ‘reformed’ informal sector may represent an important additional domestic resource for provision of services in LICs to achieve UHC.

As health regulatory bodies in resource-scarce settings have limited capacity to measure and regulate private sector quality and capacity, it will be essential to strengthen mechanisms for self-accreditation for informal providers, with a view to ensure a minimum quality threshold for services by profit and NFP providers. As suggested by a recent WHO report on engaging the private sector through improving public sector governance (World Health Organisation, 2020), engagement with private providers will necessarily have to go through collection and analysis of data to align priorities for action. This would entail: working together to achieve shared public health objectives; developing an institutional framework to empower actors and developing mutual trust amongst all actors as reliable participants. However, misconceived or exploitative regulatory provisions always carry the risk of keeping informal providers outside the formal market (Sudhinaraset *et al.*, 2013). Therefore, attempts to reform the sector will need to ponder the possible UHC gains against the risks of killing fragile providers.

## Conclusion

UHC is the current dominant paradigm in health system research. However, it is unclear to what extent formal and informal private providers contribute to the achievement of UHC in LICs. We used an original adapted UHC framework to systematically scope the evidence on private sectors and progress in universal coverage in 27 LICs from the last 20 years.

We noticed that boundaries between public, formal and informal private providers are often blurred in the academic literature, and the quality of services not always explored in the available studies on UHC. We found evidence that most primary care, mother, child and malaria services are already made available by non-public providers across all LICs. Formal private providers appear to mostly operate in well-served urban settings, while informal and not-for-profit ones cater for the lower end of the health market. However, informal providers often provide services of suboptimal quality, and fail to advance financial protection against ill health. Scarce local capacity to regulate, enforce quality and manage contracts make it also cumbersome to include in national UHC strategies. In addition, mechanisms for (self) accreditation will need to be strengthened to guarantee a minimum level of service quality from these actors.

We conclude that quality and effectiveness should be considered a core principle of universal coverage and represent an additional dimension of the UHC cube in its own right. Given their ubiquitous nature and diversity of contributions, improving the quality and financing of informal providers may also be key to expand UHC in LICs.

## Supplementary Material

czad075_Supp

## Reflexivity Statement

The authors include one lead female and two males and span multiple levels of seniority. While two of the authors specialize in global health and health financing, the third is a sociology specialist with specific public health expertise in Uganda and the East Africa region. All three authors have extensive experience conducting systematic reviews in low- and middle-income countries.
