# Supplementary material for: Do private health providers help achieve Universal Health Coverage? A scoping review of the evidence from low-income countries
Source: Health Policy Plan. 2023 Aug 21;38(9):1050–63. doi: 10.1093/heapol/czad075 (PMC10566321; doi:10.1093/heapol/czad075)
Supplement: czad075_Supp [file czad075_supp.zip › Appendix 1_Search strategy.docx]

Appendix 1 – Search strategy

**PubMed** 24/6/22 **471**

Timespan: 2002-2022

|  | **Search components** | **Results** |
| --- | --- | --- |
| 1 | private sector OR private provider OR for profit OR for-profit OR private practice OR private hospital OR private clinic OR private enterprise OR private health service OR Private healthcare OR privat* OR "hospitals, private"[MeSH Terms] OR "Private Sector"[MeSH Terms] OR "Private Practice"[MeSH Terms] OR Informal sector OR Hawker* OR Traditional medic* | 432,458 |
| 2 | universal health coverage OR UHC OR universal coverage OR universal access OR universal health* OR (Universal health care[MeSH]) | 50,328 |
| 3 | developing Countries [Mesh] OR low income countr* OR low-income countr* OR LIC OR least developed countr* | 87,272 |
| 4 | Afghanistan OR Burkina Faso OR Burundi OR "Central African Republic" OR Chad OR "Democratic Republic of Congo" OR Eritrea OR Ethiopia OR Gambia OR Guinea OR Guinea-Bissau OR "North Korea" OR Liberia OR Madagascar OR Malawi OR Mali OR Mozambique OR Niger OR Rwanda OR "Sierra Leone" OR Somalia OR "South Sudan" OR Sudan OR Syria OR Togo OR Uganda OR Yemen | 165,410 |
| 5 | 3 OR 4 | 241,765 |
| 6 | 1 AND 2 AND 5 | 568 |

(((((((((((((((Private sector) OR (private provider)) OR (for profit)) OR (for-profit)) OR (private practice)) OR (private hospital)) OR (private clinic)) OR (private enterprise)) OR (private health service)) OR (Private healthcare)) OR (privat*) OR "hospitals, private"[MeSH Terms] OR "Private Sector"[MeSH Terms] OR "Private Practice"[MeSH Terms] ) OR (Informal sector[Title/Abstract])) OR (Hawker*[Title/Abstract])) OR (Traditional medic*[Title/Abstract]) **AND** (2002:3000/12/12[pdat])) **AND** ((universal health coverage OR UHC OR universal coverage OR universal access OR universal health care OR universal healthcare) OR (Universal health care[MeSH]) AND (2002:3000/12/12[pdat]))) **AND** ("Developing Countries"[Mesh] OR low income countr* OR LIC OR Least Developed Countr* OR Afghanistan OR Burkina Faso OR Burundi OR "Central African Republic" OR Chad OR "Democratic Republic of Congo" OR Eritrea OR Ethiopia OR Gambia OR Guinea OR Guinea-Bissau OR "North Korea" OR Liberia OR Madagascar OR Malawi OR Mali OR Mozambique OR Niger OR Rwanda OR "Sierra Leone" OR Somalia OR "South Sudan" OR Sudan OR Syria OR Togo OR Uganda OR Yemen AND (2002:3000/12/12[pdat]))

((private sector OR private provider OR for profit OR for-profit OR private practice OR private hospital OR private clinic OR private enterprise OR private health service OR Private healthcare OR privat* OR "hospitals, private"[MeSH Terms] OR "Private Sector"[MeSH Terms] OR "Private Practice"[MeSH Terms] OR Informal sector OR Hawker* OR Traditional medic* AND (2002:3000/12/12[pdat])) AND (universal health coverage OR UHC OR universal coverage OR universal access OR universal health* OR (Universal health care[MeSH]) AND (2002:3000/12/12[pdat]))) AND ((Afghanistan OR Burkina Faso OR Burundi OR "Central African Republic" OR Chad OR "Democratic Republic of Congo" OR Eritrea OR Ethiopia OR Gambia OR Guinea OR Guinea-Bissau OR "North Korea" OR Liberia OR Madagascar OR Malawi OR Mali OR Mozambique OR Niger OR Rwanda OR "Sierra Leone" OR Somalia OR "South Sudan" OR Sudan OR Syria OR Togo OR Uganda OR Yemen AND (2002:3000/12/12[pdat])) OR (developing Countries [Mesh] OR low income countr* OR low-income countr* OR LIC OR least developed countr* AND (2002:3000/12/12[pdat])) AND (2002:3000/12/12[pdat])) - Saved search Filters: from 2002 - 3000/12/12

(("Private Sector"[MeSH Terms] OR ("private"[All Fields] AND "sector"[All Fields]) OR "Private Sector"[All Fields] OR (("patients rooms"[MeSH Terms] OR ("patients"[All Fields] AND "rooms"[All Fields]) OR "patients rooms"[All Fields] OR "private"[All Fields] OR "privately"[All Fields] OR "privates"[All Fields] OR "privatization"[MeSH Terms] OR "privatization"[All Fields] OR "privatizations"[All Fields] OR "privatize"[All Fields] OR "privatized"[All Fields] OR "privatizing"[All Fields]) AND ("provide"[All Fields] OR "provided"[All Fields] OR "provider"[All Fields] OR "provider s"[All Fields] OR "providers"[All Fields] OR "provides"[All Fields] OR "providing"[All Fields])) OR ("profit"[All Fields] OR "profitabilities"[All Fields] OR "profitability"[All Fields] OR "profitable"[All Fields] OR "profitableness"[All Fields] OR "profitably"[All Fields] OR "profiteering"[All Fields] OR "profiteers"[All Fields] OR "profiting"[All Fields] OR "profits"[All Fields]) OR "for-profit"[All Fields] OR ("Private Practice"[MeSH Terms] OR ("private"[All Fields] AND "practice"[All Fields]) OR "Private Practice"[All Fields]) OR ("hospitals, private"[MeSH Terms] OR ("hospitals"[All Fields] AND "private"[All Fields]) OR "private hospitals"[All Fields] OR ("private"[All Fields] AND "hospital"[All Fields]) OR "private hospital"[All Fields]) OR (("patients rooms"[MeSH Terms] OR ("patients"[All Fields] AND "rooms"[All Fields]) OR "patients rooms"[All Fields] OR "private"[All Fields] OR "privately"[All Fields] OR "privates"[All Fields] OR "privatization"[MeSH Terms] OR "privatization"[All Fields] OR "privatizations"[All Fields] OR "privatize"[All Fields] OR "privatized"[All Fields] OR "privatizing"[All Fields]) AND ("ambulatory care facilities"[MeSH Terms] OR ("ambulatory"[All Fields] AND "care"[All Fields] AND "facilities"[All Fields]) OR "ambulatory care facilities"[All Fields] OR "clinic"[All Fields] OR "clinic s"[All Fields] OR "clinical"[All Fields] OR "clinically"[All Fields] OR "clinicals"[All Fields] OR "clinics"[All Fields])) OR ("Private Sector"[MeSH Terms] OR ("private"[All Fields] AND "sector"[All Fields]) OR "Private Sector"[All Fields] OR ("private"[All Fields] AND "enterprise"[All Fields]) OR "private enterprise"[All Fields]) OR (("patients rooms"[MeSH Terms] OR ("patients"[All Fields] AND "rooms"[All Fields]) OR "patients rooms"[All Fields] OR "private"[All Fields] OR "privately"[All Fields] OR "privates"[All Fields] OR "privatization"[MeSH Terms] OR "privatization"[All Fields] OR "privatizations"[All Fields] OR "privatize"[All Fields] OR "privatized"[All Fields] OR "privatizing"[All Fields]) AND ("health services"[MeSH Terms] OR ("health"[All Fields] AND "services"[All Fields]) OR "health services"[All Fields] OR ("health"[All Fields] AND "service"[All Fields]) OR "health service"[All Fields])) OR (("patients rooms"[MeSH Terms] OR ("patients"[All Fields] AND "rooms"[All Fields]) OR "patients rooms"[All Fields] OR "private"[All Fields] OR "privately"[All Fields] OR "privates"[All Fields] OR "privatization"[MeSH Terms] OR "privatization"[All Fields] OR "privatizations"[All Fields] OR "privatize"[All Fields] OR "privatized"[All Fields] OR "privatizing"[All Fields]) AND ("delivery of health care"[MeSH Terms] OR ("delivery"[All Fields] AND "health"[All Fields] AND "care"[All Fields]) OR "delivery of health care"[All Fields] OR "healthcare"[All Fields] OR "healthcare s"[All Fields] OR "healthcares"[All Fields])) OR "privat*"[All Fields] OR "hospitals, private"[MeSH Terms] OR "Private Sector"[MeSH Terms] OR "Private Practice"[MeSH Terms] OR ("informal sector"[MeSH Terms] OR ("informal"[All Fields] AND "sector"[All Fields]) OR "informal sector"[All Fields]) OR "hawker*"[All Fields] OR (("tradition"[All Fields] OR "tradition s"[All Fields] OR "traditional"[All Fields] OR "traditionals"[All Fields] OR "traditions"[All Fields]) AND "medic*"[All Fields])) AND 2002/01/01:3000/12/12[Date - Publication] AND (((("universal"[All Fields] OR "universalism"[All Fields] OR "universalities"[All Fields] OR "universality"[All Fields] OR "universalization"[All Fields] OR "universalize"[All Fields] OR "universalized"[All Fields] OR "universalizing"[All Fields] OR "universally"[All Fields] OR "universals"[All Fields] OR "universe"[All Fields] OR "universes"[All Fields]) AND ("health"[MeSH Terms] OR "health"[All Fields] OR "health s"[All Fields] OR "healthful"[All Fields] OR "healthfulness"[All Fields] OR "healths"[All Fields]) AND ("coverage"[All Fields] OR "coverages"[All Fields])) OR "UHC"[All Fields] OR ("universal health insurance"[MeSH Terms] OR ("universal"[All Fields] AND "health"[All Fields] AND "insurance"[All Fields]) OR "universal health insurance"[All Fields] OR ("universal"[All Fields] AND "coverage"[All Fields]) OR "universal coverage"[All Fields]) OR (("universal"[All Fields] OR "universalism"[All Fields] OR "universalities"[All Fields] OR "universality"[All Fields] OR "universalization"[All Fields] OR "universalize"[All Fields] OR "universalized"[All Fields] OR "universalizing"[All Fields] OR "universally"[All Fields] OR "universals"[All Fields] OR "universe"[All Fields] OR "universes"[All Fields]) AND ("access"[All Fields] OR "accessed"[All Fields] OR "accesses"[All Fields] OR "accessibilities"[All Fields] OR "accessibility"[All Fields] OR "accessible"[All Fields] OR "accessing"[All Fields])) OR (("universal"[All Fields] OR "universalism"[All Fields] OR "universalities"[All Fields] OR "universality"[All Fields] OR "universalization"[All Fields] OR "universalize"[All Fields] OR "universalized"[All Fields] OR "universalizing"[All Fields] OR "universally"[All Fields] OR "universals"[All Fields] OR "universe"[All Fields] OR "universes"[All Fields]) AND "health*"[All Fields]) OR "universal health care"[MeSH Terms]) AND 2002/01/01:3000/12/12[Date - Publication]) AND (((("afghanistan"[MeSH Terms] OR "afghanistan"[All Fields] OR "afghanistan s"[All Fields] OR ("burkina faso"[MeSH Terms] OR ("burkina"[All Fields] AND "faso"[All Fields]) OR "burkina faso"[All Fields]) OR ("burundi"[MeSH Terms] OR "burundi"[All Fields]) OR "Central African Republic"[All Fields] OR ("chad"[MeSH Terms] OR "chad"[All Fields]) OR "Democratic Republic of Congo"[All Fields] OR ("eritrea"[MeSH Terms] OR "eritrea"[All Fields]) OR ("ethiopia"[MeSH Terms] OR "ethiopia"[All Fields] OR "ethiopia s"[All Fields]) OR ("gambia"[MeSH Terms] OR "gambia"[All Fields] OR "gambia s"[All Fields]) OR ("guinea"[MeSH Terms] OR "guinea"[All Fields] OR "guinea s"[All Fields] OR "guineas"[All Fields]) OR ("guinea bissau"[MeSH Terms] OR "guinea bissau"[All Fields] OR ("guinea"[All Fields] AND "bissau"[All Fields]) OR "guinea bissau"[All Fields]) OR "North Korea"[All Fields] OR ("liberia"[MeSH Terms] OR "liberia"[All Fields] OR "liberia s"[All Fields]) OR ("madagascar"[MeSH Terms] OR "madagascar"[All Fields] OR "madagascar s"[All Fields]) OR ("malawi"[MeSH Terms] OR "malawi"[All Fields] OR "malawi s"[All Fields]) OR ("mali"[MeSH Terms] OR "mali"[All Fields]) OR ("mozambique"[MeSH Terms] OR "mozambique"[All Fields] OR "mozambique s"[All Fields]) OR ("niger"[MeSH Terms] OR "niger"[All Fields]) OR ("rwanda"[MeSH Terms] OR "rwanda"[All Fields] OR "rwanda s"[All Fields]) OR "Sierra Leone"[All Fields] OR ("somalia"[MeSH Terms] OR "somalia"[All Fields]) OR "South Sudan"[All Fields] OR ("sudan"[MeSH Terms] OR "sudan"[All Fields] OR "sudans"[All Fields] OR "sudan s"[All Fields]) OR ("syria"[MeSH Terms] OR "syria"[All Fields] OR "syria s"[All Fields]) OR ("togo"[MeSH Terms] OR "togo"[All Fields]) OR ("uganda"[MeSH Terms] OR "uganda"[All Fields] OR "uganda s"[All Fields]) OR ("yemen"[MeSH Terms] OR "yemen"[All Fields])) AND 2002/01/01:3000/12/12[Date - Publication]) OR (("developing countries"[MeSH Terms] OR (("poverty"[MeSH Terms] OR "poverty"[All Fields] OR ("low"[All Fields] AND "income"[All Fields]) OR "low income"[All Fields]) AND "countr*"[All Fields]) OR (("poverty"[MeSH Terms] OR "poverty"[All Fields] OR ("low"[All Fields] AND "income"[All Fields]) OR "low income"[All Fields]) AND "countr*"[All Fields]) OR "LIC"[All Fields] OR ("least"[All Fields] AND ("develop"[All Fields] OR "develope"[All Fields] OR "developed"[All Fields] OR "developer"[All Fields] OR "developer s"[All Fields] OR "developers"[All Fields] OR "developing"[All Fields] OR "developments"[All Fields] OR "develops"[All Fields] OR "growth and development"[MeSH Subheading] OR ("growth"[All Fields] AND "development"[All Fields]) OR "growth and development"[All Fields] OR "development"[All Fields]) AND "countr*"[All Fields])) AND 2002/01/01:3000/12/12[Date - Publication])) AND 2002/01/01:3000/12/12[Date - Publication])) AND (2002:3000/12/12[pdat])

**Web of Science – core collection** 24/6/22 **622**

Timespan: 2002-2022

|  | **Search components** | **Results** |
| --- | --- | --- |
| 1 | ((((((((((((((TS=(private sector)) OR TS=(private provider )) OR TS=(for profit)) OR TS=(for-profit)) OR TS=(private practice)) OR TS=(private hospital)) OR TS=(private clinic)) OR TS=(private enterprise)) OR TS=(private health service)) OR TS=(private healthcare)) OR TS=(privat*)) OR TS=(informal sector)) OR TS=(informal provider)) OR TS=(hawker*)) OR TS=(traditional medic*) | 530,927 |
| 2 | universal health coverage OR UHC OR universal coverage OR universal access OR universal health* | 43,123 |
| 3 | developing countr* OR low income countr* OR low-income countr* OR LIC OR least developed countr* | 382,420 |
| 4 | Afghanistan OR “Burkina Faso” OR Burundi OR "Central African Republic" OR Chad OR "Democratic Republic of Congo" OR Eritrea OR Ethiopia OR Gambia OR Guinea OR Guinea-Bissau OR "North Korea" OR Liberia OR Madagascar OR Malawi OR Mali OR Mozambique OR Niger OR Rwanda OR "Sierra Leone" OR Somalia OR "South Sudan" OR Sudan OR Syria OR Togo OR Uganda OR Yemen | 375,993 |
| 5 | 3 OR 4 | 738,361 |
| 6 | 1 AND 2 AND 5 | 622 |

**Scopus** 24/6/22 **620**

Timespan: 2002-present

|  | **Search components** | **Results** |
| --- | --- | --- |
| 1 | ( TITLE-ABS-KEY ( private  AND sector )  OR  TITLE-ABS-KEY ( private  AND provider )  OR  TITLE-ABS-KEY ( for  AND profit )  OR  TITLE-ABS-KEY ( for-profit )  OR  TITLE-ABS-KEY ( private  AND practice )  OR  TITLE-ABS-KEY ( private  AND hospital )  OR  TITLE-ABS-KEY ( private  AND health* )  OR  TITLE-ABS-KEY ( informal  AND sector )  OR  TITLE-ABS-KEY ( informal  AND provider* )  OR  TITLE-ABS-KEY ( hawker* )  OR  TITLE-ABS-KEY ( traditional  AND medic* )  OR  TITLE-ABS-KEY ( private  AND enterprise ) )  AND  PUBYEAR  >  2001  AND  PUBYEAR  >  2001 | 542,826 |
| 2 | ( TITLE-ABS-KEY ( universal  AND health  AND coverage )  OR  TITLE-ABS-KEY ( uhc )  OR  TITLE-ABS-KEY ( universal  AND coverage )  OR  TITLE-ABS-KEY ( universal  AND access )  OR  TITLE-ABS-KEY ( universal  AND health* ) )  AND  PUBYEAR  >  2001  AND  PUBYEAR  >  2001 | 54,545 |
| 3 | ( TITLE-ABS-KEY ( developing  AND countr* )  OR  TITLE-ABS-KEY ( low  AND income  AND countr* )  OR  TITLE-ABS-KEY ( low-income  AND countr* )  OR  TITLE-ABS-KEY ( lic )  OR  TITLE-ABS-KEY ( least  AND developed  AND countr* ) )  AND  PUBYEAR  >  2001  AND  PUBYEAR  >  2001 | 310,373 |
| 4 | ( TITLE-ABS-KEY ( afghanistan )  OR  TITLE-ABS-KEY ( "Burkina Faso" )  OR  TITLE-ABS-KEY ( burundi )  OR  TITLE-ABS-KEY ( "Central African Republic" )  OR  TITLE-ABS-KEY ( chad )  OR  TITLE-ABS-KEY ( "Democratic Republic of Congo" )  OR  TITLE-ABS-KEY ( drc )  OR  TITLE-ABS-KEY ( eritrea )  OR  TITLE-ABS-KEY ( ethiopia )  OR  TITLE-ABS-KEY ( gambia )  OR  TITLE-ABS-KEY ( guinea )  OR  TITLE-ABS-KEY ( guinea-bissau )  OR  TITLE-ABS-KEY ( "North Korea" )  OR  TITLE-ABS-KEY ( liberia )  OR  TITLE-ABS-KEY ( madagascar )  OR  TITLE-ABS-KEY ( malawi )  OR  TITLE-ABS-KEY ( mali )  OR  TITLE-ABS-KEY ( mozambique )  OR  TITLE-ABS-KEY ( niger )  OR  TITLE-ABS-KEY ( rwanda )  OR  TITLE-ABS-KEY ( "Sierra Leone" )  OR  TITLE-ABS-KEY ( somalia )  OR  TITLE-ABS-KEY ( "South Sudan" )  OR  TITLE-ABS-KEY ( sudan )  OR  TITLE-ABS-KEY ( syria )  OR  TITLE-ABS-KEY ( togo )  OR  TITLE-ABS-KEY ( uganda )  OR  TITLE-ABS-KEY ( yemen ) )  AND  PUBYEAR  >  2001  AND  PUBYEAR  >  2001 | 290,752 |
| 5 | 3 OR 4 | 582,807 |
| 6 | ( ( TITLE-ABS-KEY ( private  AND sector )  OR  TITLE-ABS-KEY ( private  AND provider )  OR  TITLE-ABS-KEY ( for  AND profit )  OR  TITLE-ABS-KEY ( for-profit )  OR  TITLE-ABS-KEY ( private  AND practice )  OR  TITLE-ABS-KEY ( private  AND hospital )  OR  TITLE-ABS-KEY ( private  AND health* )  OR  TITLE-ABS-KEY ( informal  AND sector )  OR  TITLE-ABS-KEY ( informal  AND provider* )  OR  TITLE-ABS-KEY ( hawker* )  OR  TITLE-ABS-KEY ( traditional  AND medic* )  OR  TITLE-ABS-KEY ( private  AND enterprise ) )  AND  PUBYEAR  >  2001  AND  PUBYEAR  >  2001 )  AND  ( ( TITLE-ABS-KEY ( universal  AND health  AND coverage )  OR  TITLE-ABS-KEY ( uhc )  OR  TITLE-ABS-KEY ( universal  AND coverage )  OR  TITLE-ABS-KEY ( universal  AND access )  OR  TITLE-ABS-KEY ( universal  AND health* ) )  AND  PUBYEAR  >  2001  AND  PUBYEAR  >  2001 )  AND  ( ( ( TITLE-ABS-KEY ( developing  AND countr* )  OR  TITLE-ABS-KEY ( low  AND income  AND countr* )  OR  TITLE-ABS-KEY ( low-income  AND countr* )  OR  TITLE-ABS-KEY ( lic )  OR  TITLE-ABS-KEY ( least  AND developed  AND countr* ) )  AND  PUBYEAR  >  2001  AND  PUBYEAR  >  2001 )  OR  ( ( TITLE-ABS-KEY ( afghanistan )  OR  TITLE-ABS-KEY ( "Burkina Faso" )  OR  TITLE-ABS-KEY ( burundi )  OR  TITLE-ABS-KEY ( "Central African Republic" )  OR  TITLE-ABS-KEY ( chad )  OR  TITLE-ABS-KEY ( "Democratic Republic of Congo" )  OR  TITLE-ABS-KEY ( drc )  OR  TITLE-ABS-KEY ( eritrea )  OR  TITLE-ABS-KEY ( ethiopia )  OR  TITLE-ABS-KEY ( gambia )  OR  TITLE-ABS-KEY ( guinea )  OR  TITLE-ABS-KEY ( guinea-bissau )  OR  TITLE-ABS-KEY ( "North Korea" )  OR  TITLE-ABS-KEY ( liberia )  OR  TITLE-ABS-KEY ( madagascar )  OR  TITLE-ABS-KEY ( malawi )  OR  TITLE-ABS-KEY ( mali )  OR  TITLE-ABS-KEY ( mozambique )  OR  TITLE-ABS-KEY ( niger )  OR  TITLE-ABS-KEY ( rwanda )  OR  TITLE-ABS-KEY ( "Sierra Leone" )  OR  TITLE-ABS-KEY ( somalia )  OR  TITLE-ABS-KEY ( "South Sudan" )  OR  TITLE-ABS-KEY ( sudan )  OR  TITLE-ABS-KEY ( syria )  OR  TITLE-ABS-KEY ( togo )  OR  TITLE-ABS-KEY ( uganda )  OR  TITLE-ABS-KEY ( yemen ) )  AND  PUBYEAR  >  2001  AND  PUBYEAR  >  2001 ) ) | 620 |

Total = 1810
