# Supplementary material for: Do private health providers help achieve Universal Health Coverage? A scoping review of the evidence from low-income countries
Source: Health Policy Plan. 2023 Aug 21;38(9):1050–63. doi: 10.1093/heapol/czad075 (PMC10566321; doi:10.1093/heapol/czad075)
Supplement: czad075_Supp [file czad075_supp.zip › Appendix 2_Articles included and excluded, with reason.docx]

Appendix 2_Articles included and excluded after full text screening, with reason

| **Title** | **Authors** | **Year** | **Journal** | **Incl/Excl** | **Reason** |
| --- | --- | --- | --- | --- | --- |
| Achieving high coverage in Rwanda's national human papillomavirus vaccination programme | Binagwaho A, Wagner CM, Gatera M, Karema C, Nutt CT, Ngabo F. | 2012 | Bull World Health Organ | Exclude | Editorial style piece; not a research article |
| Urban Health Extension Program and Health Services Utilization in Northwest Ethiopia: A Community-Based Study | Molla S, Tsehay CT, Gebremedhin T. | 2020 | Risk Manag Healthc Policy | Exclude | Not private sector or provider |
| A comprehensive assessment of universal health coverage in 111 countries: a retrospective observational study | Wagstaff A, Neelsen S. | 2020 | Lancet Glob Health | Exclude | Does not address private sector or providers |
| Business models for primary health care delivery in low- and middle-income countries: a scoping study of nine social entrepreneurs | Lokman L, Chahine T. | 2021 | BMC Health Serv Res | Exclude | Not specific to LICs |
| Effect of donor funding for immunization from Gavi and other development assistance channels on vaccine coverage: Evidence from 120 low and middle income recipient countries | Ikilezi G., Augusto O.J., Dieleman J.L., Sherr K., Lim S.S. | 2020 | Vaccine | Exclude | Not specific to private sector or providers |
| Effect of mobile application user interface improvements on minimum expected home visit coverage by community health workers in Mali: a randomised controlled trial | Yang JE, Lassala D, Liu JX, Whidden C, Holeman I, Keita Y, Djiguiba Y, N'Diaye SI, Fall F, Kayentao K, Johnson AD. | 2021 | BMJ Glob Health | Exclude | Not private sector or provider |
| Financing mental health services in low- and middle-income countries | Dixon A, McDaid D, Knapp M, Curran C. | 2006 | Health Policy Plan | Exclude | Not specific to LICs |
| Formal and informal medicine retailers in Sub-Saharan Africa: a scoping review of research trends | Oleffe A, Sako B, Paul E, Mahieu C. | 2022 | Int J Pharm Pract | Exclude | Not specific to LICs |
| Introducing malaria rapid diagnostic tests in private medicine retail outlets: A systematic literature review | Visser T, Bruxvoort K, Maloney K, Leslie T, Barat LM, Allan R, Ansah EK, Anyanti J, Boulton I, Clarke SE, Cohen JL, Cohen JM, Cutherell A, Dolkart C, Eves K, Fink G, Goodman C, Hutchinson E, Lal S, Mbonye A, Onwujekwe O, Petty N, Pontarollo J, Poyer S, Schellenberg D, Streat E, Ward A, Wiseman V, Whitty CJ, Yeung S, Cunningham J, Chandler CI. | 2017 | PLoS One | Exclude | Not specific to LICs |
| Measurement and analysis of inequality of opportunity in access of maternal and child health care in Togo | Sanoussi Y. | 2017 | BMC Health Serv Res | Exclude | Not specific to private sector or providers |
| Regulating private health insurance to serve the public interest: policy issues for developing countries | Sekhri N, Savedoff W. | 2006 | Int J Health Plann Manage | Exclude | Not systematic review |
| The failure of private health services: COVID-19 induced crises in low- and middle-income country (LMIC) health systems | David Williams O, Yung KC, Grépin KA. | 2021 | Glob Public Health | Exclude | Not specific to LICs |
| The impact of health insurance in Africa and Asia: a systematic review | Spaan E, Mathijssen J, Tromp N, McBain F, ten Have A, Baltussen R. | 2012 | Bull World Health Organ | Exclude | Not specific to LICs |
| Trends in future health financing and coverage: future health spending and universal health coverage in 188 countries, 2016-40 | Global Burden of Disease Health Financing Collaborator Network. | 2018 | Lancet | Exclude | Not specific to LICs and private sector |
| Private health insurance: implications for developing countries | Sekhri N, Savedoff W. | 2005 | Bull World Health Organ | Exclude | Not LIC specific |
| 'They say we are money minded' exploring experiences of formal private for-profit health providers towards contribution to pro-poor access in post conflict Northern Uganda | Namakula J, Fustukian S, McPake B, Ssengooba F. | 2021 | Glob Health Action | Include | Mixed methods data on for-profit health providers expanding access to poor populations in Uganda |
| Availability, pricing and affordability of essential medicines in Eastern Ethiopia: a comprehensive analysis using WHO/HAI methodology | Sisay M, Amare F, Hagos B, Edessa D. | 2021 | J Pharm Policy Pract | Include | Quant data on availability and affordabilty of essential medicines in Ethiopia disaggregated by private vs public sector |
| Does the type of health insurance enrollment affect provider choice, utilization and health care expenditures? | Atake EH. | 2020 | BMC Health Serv Res | Include | Quant data on effect of private v public insurance on health service use and financial protection/CHE in Togo |
| Exploring health insurance services in Sudan from the perspectives of insurers | Salim AMA, Hamed FHM. | 2018 | SAGE Open Med | Include | Qual data on perceptions of private & public insurance contribution to UHC/financial protection in Sudan |
| Local experience of using traditional medicine in northern Rwanda: a qualitative study | Tan M, Otake Y, Tamming T, Akuredusenge V, Uwinama B, Hagenimana F. | 2021 | BMC Complement Med Ther | Include | Qual interview and obs data on the role of traditional medicine providers in expanding UHC coverage and financial protection in Rwanda |
| Out-of-pocket expenditure and its determinants in the context of private healthcare sector expansion in sub-Saharan Africa urban cities: evidence from household survey in Ouagadougou, Burkina Faso | Beogo I, Huang N, Gagnon MP, Amendah DD. | 2016 | BMC Res Notes | Include | Quant data on OOP expenditure for public v private acute care in Burkina |
| Promoting universal financial protection: contracting faith-based health facilities to expand access - lessons learned from Malawi | Chirwa, ML; Kazanga, I; Faedo, G; Thomas, S | 2013 | HEALTH RESEARCH POLICY AND SYSTEMS | Include | Mixed methods/policy analysis on role of faith-based private providers in expadning financial risk protection in Malawi |
| Sources of Health Care Among Under-5 Malawian Children With Diarrhea Episodes: An Analysis of the 2017 Demographic and Health Survey | Nyasulu PS, Ngamasana E, Kandala NB. | 2019 | Glob Pediatr Health | Include | Quant data on private v public provision of diarrhea treatment to under 5s in urban/rural Malawi |
| Strengthening close to community provision of maternal health services in fragile settings: an exploration of the changing roles of TBAs in Sierra Leone and Somaliland | Orya E, Adaji S, Pyone T, Wurie H, van den Broek N, Theobald S. | 2017 | BMC Health Serv Res | Include | Qual interview data on role of traditional birth attendants in increasing coverage of maternal health servives in Sierra Leone and Somaliland |
| Time trends in facility-based and private-sector childbirth care: analysis of Demographic and Health Surveys from 25 sub-Saharan African countries from 2000 to 2016 | Doctor HV, Radovich E, Benova L. | 2019 | J Glob Health | Include | Analysis of household survey data on private & public provision of childbirth services by wealth in SSA - disaggregated by country - including 12 LICs |
| Two decades of antenatal and delivery care in Uganda: a cross-sectional study using Demographic and Health Surveys | Benova L, Dennis ML, Lange IL, Campbell OMR, Waiswa P, Haemmerli M, Fernandez Y, Kerber K, Lawn JE, Santos AC, Matovu F, Macleod D, Goodman C, Penn-Kekana L, Ssengooba F, Lynch CA. | 2018 | BMC Health Serv Res | Include | Household survey data on private provision of antenatal care coverage in Uganda |
| Use of family planning and child health services in the private sector: an equity analysis of 12 DHS surveys | Chakraborty NM, Sprockett A. | 2018 | Int J Equity Health | Include | Secondary analysis of household survey data on private provision of maternal/child services by wealth in SSA - disaggregated by country, includes 3 LICs |
| Utilization of HIV-related services from the private health sector: A multi-country analysis | Wang, WJ; Sulzbach, S; De, S | 2011 | SOCIAL SCIENCE & MEDICINE | Include | Analysis of survey data on private v public provision of HIV services in SSA - disagg by country - includes 5 LICs |
| Abolition of user fees: the Uganda paradox | Nabyonga Orem J, Mugisha F, Kirunga C, Macq J, Criel B. | 2011 | Health Policy Plan | Include | Analysis of household data on use and expenditure on private & public clinics in Uganda by wealth |
| An audit of registered radiology equipment resources in Uganda | Kiguli-Malwadde E, Byanyima R, Kawooya MG, Mubuuke AG, Basiimwa RC, Pitcher R. | 2020 | Pan Afr Med J | Include | Analysis of data on distribution/coverage and public/private ownership of radiology equipment in Uganda |
| An equity analysis of utilization of health services in Afghanistan using a national household survey | Kim C, Saeed KM, Salehi AS, Zeng W. | 2016 | BMC Public Health | Include | Quant data on use of public & private inpatient/OP/maternity services in Afganistan by wealth quiintile |
| Availability and affordability of antimalarial and antibiotic medicines in Malawi | Khuluza F, Heide L. | 2017 | PLoS One | Include | Quant data on availability and affordability of malarial medicine provided by private v public sector in Malawi |
| Availability and price of malaria rapid diagnostic tests in the public and private health sectors in 2011: results from 10 nationally representative cross-sectional retail surveys | Poyer S, Shewchuk T, Tougher S, Ye Y; ACTwatch Group, Mann AG, Willey BA, Thomson R, Amuasi JH, Ren R, Wamukoya M, Taylor M, Nguah SB, Mberu B, Kalolella A, Juma E, Festo C, Johanes B, Diap G, Bruxvoort K, Ansong D, Hanson K, Arnold F, Goodman C. | 2015 | Trop Med Int Health | Include | Quant data on malaria diagnostic market share by public/private providers in 9 LMICs - disaggregated including 2 LICs |
| Beneficiaries of conflict: a qualitative study of people's trust in the private health care system in Mogadishu, Somalia | Gele AA, Ahmed MY, Kour P, Moallim SA, Salad AM, Kumar B. | 2017 | Risk Manag Healthc Policy | Include | Qual interviews on accessibility and affordability of private health centres in Somalia |
| Commercialization of obstetric and neonatal care in the Democratic Republic of the Congo: A study of the variability in user fees in Lubumbashi, 2014 | Ntambue A.M., Malonga F.K., Dramaix-Wilmet M., Ilunga T.M., Musau A.N., Matungulu C.M., Cowgill K.D., Donnen P. | 2018 | PLoS ONE | Include | Quant data on user fees/OOPP to private v public maternal services in DRC |
| Gaps in universal health coverage in Malawi: a qualitative study in rural communities | Abiiro GA, Mbera GB, De Allegri M. | 2014 | BMC Health Serv Res | Include | Qual interview data on percieved gaps in population coverage and financial protection by private and public providers in rural Malawi |
| Government resource contributions to the private-not-for-profit sector in Uganda: evolution, adaptations and implications for universal health coverage | Ssennyonjo A, Namakula J, Kasyaba R, Orach S, Bennett S, Ssengooba F. | 2018 | Int J Equity Health | Include | Mixed methods/case study on a public-private partnership in Uganda and its effects on user fees and coverage |
| Malaria related care-seeking-behaviour and expenditures in urban settings: A household survey in Ouagadougou, Burkina Faso | Beogo I, Huang N, Drabo MK, Yé Y. | 2016 | Acta Trop | Include | Quant household survey data on use of and OOPP to public & private malaria treatment in Burkina |
| Voluntary Health Insurance expenditure in low- and middle-income countries: Exploring trends during 1995-2012 and policy implications for progress towards universal health coverage | Pettigrew LM, Mathauer I. | 2016 | Int J Equity Health | Include | Quant data on trends in private insurance expenditure by country income group - some individual LICs disaggregated |
| A public-private partnership for dialysis provision in Ethiopia: a model for high-cost care in low-resource settings | Paltiel O, Berhe E, Aberha AH, Tequare MH, Balabanova D. | 2020 | Health Policy Plan | Exclude | Report does not meet evidence criteria |
| Are social franchises contributing to universal access to reproductive health services in low-income countries? | Sundari Ravindran TK, Fonn S. | 2011 | Reprod Health Matters | Include | Quant & qual data on affect of private provider networks on expanding UHC in LICs and LMICs - disaggregated by country |
| Baseline assessment of WHO's target for both availability and affordability of essential medicines to treat non-communicable diseases | Ewen M, Zweekhorst M, Regeer B, Laing R. | 2017 | PLoS One | Include | Quant data on public v private provision of affordable NCD meds in LICs and MICs - compares contribution to WHO target. LICs at time of research - unsure meets context criteria |
| Engagement of non-governmental organisations in moving towards universal health coverage: a scoping review | Sanadgol, A; Doshmangir, L; Majdzadeh, R; Gordeev, VS | 2021 | GLOBALIZATION AND HEALTH | Exclude | Not specific to LICs |
| Getting to FP2020: Harnessing the private sector to increase modern contraceptive access and choice in Ethiopia, Nigeria, and DRC | Riley C, Garfinkel D, Thanel K, Esch K, Workalemahu E, Anyanti J, Mpanya G, Binanga A, Pope J, Longfield K, Bertrand J, Shaw B; FPwatch Group. | 2018 | PLoS One | Include | Quant data on private v public sector market share and provision/affordability of contraceptives in Ethiopia, Nigeria, and DRC (two LICs, one LMIC) - data disagregated by country. |
| Malaria, medicines and miles: A novel approach to measuring access to treatment from a household perspective | Palafox B, Goodman C, Hanson K. | 2019 | SSM Popul Health | Include | Quant data on urban and rural household antimalarial coverage population by public & private providers in Benin, Nigeria, Uganda and Zambia |
| Nature of the private hospital services toward universal health coverage: A systematic scoping review of the developing countries evidence | Fallah, R; Maleki, M | 2021 | JOURNAL OF EDUCATION AND HEALTH PROMOTION | Exclude | Not specific to LICs |
| Participation of delivering private hospital services in universal health coverage: A systematic scoping review of the developing countries' evidence | Fallah R, Bazrafshan A. | 2021 | J Educ Health Promot | Exclude | Not specific to LICs |
| Private Sector An Important But Not Dominant Provider Of Key Health Services In Low- And Middle-Income Countries | Grépin KA. | 2016 | Health Aff (Millwood) | Include | Secondary analysis of Demographic and Health Survey data on private sector provision of selected health services in LMICs - disaggregates use in LIC/lowerMIC/upperMIC |
| Testing times: trends in availability, price, and market share of malaria diagnostics in the public and private healthcare sector across eight sub-Saharan African countries from 2009 to 2015 | ACTwatch Group, Hanson K, Goodman C. | 2017 | Malar J | Include | Quant survey data on private & public sector market share/provision of malaria services for 8 individual SSA countries - includes DRC, Uganda & Madagascar, as well as several LMICs |
| Family planning, antenatal and delivery care: cross-sectional survey evidence on levels of coverage and inequalities by public and private sector in 57 low- and middle-income countries | Campbell OM, Benova L, MacLeod D, Baggaley RF, Rodrigues LC, Hanson K, Powell-Jackson T, Penn-Kekana L, Polonsky R, Footman K, Vahanian A, Pereira SK, Santos AC, Filippi VG, Lynch CA, Goodman C. | 2016 | Trop Med Int Health | Include | Quant data on private sector coverage of maternal/newborn health service in LMICs - disaggregates LICs |
| Factors enabling comprehensive maternal health services in the benefits package of emerging financing schemes: A cross-sectional analysis from 1990 to 2014 | Vargas V, Ahmed S, Adams AM. | 2018 | PLoS One | Exclude | Not specific to LICs |
| Utilization of traditional medicine in primary health care in low- and middle-income countries: a systematic review | Kim JK, Kim KH, Shin YC, Jang BH, Ko SG. | 2020 | Health Policy Plan | Exclude | Not specific to LICs |
| A qualitative inquiry of access to and quality of primary healthcare in seven communities in East and West Africa (SevenCEWA): perspectives of stakeholders, healthcare providers and users | Ameh S, Akeem BO, Ochimana C, Oluwasanu AO, Mohamed SF, Okello S, Muhihi A, Danaei G. | 2021 | BMC Fam Pract | Include | Qual interview data on PHC coverage in East and West Africa - some specific data on the LIC included but generally takes the LMICs together |
| Engagement of the private pharmaceutical sector for TB control: rhetoric or reality? | Konduri N, Delmotte E, Rutta E. | 2017 | J Pharm Policy Pract | Exclude | Not specific to LICs |
| Leveraging the lessons learned from financing HIV programs to advance the universal health coverage (UHC) agenda in the East African Community | Zakumumpa H, Bennett S, Ssengooba F. | 2019 | Glob Health Res Policy | Exclude | Not specific to LICs |
| Prohibit, constrain, encourage, or purchase: how should we engage with the private health-care sector? | Montagu D, Goodman C. | 2016 | Lancet | Exclude | Based on a systematic review but doesn't report it, not specific to LICs |
| Standard Survey Data: Insights Into Private Sector Utilization | Montagu D, Chakraborty N. | 2021 | Front Med (Lausanne) | Include | Quant data on private sector provision of inpatient/OP care by WHO region - includes some country-specific data |
| Towards universal health coverage: advancing the development and use of traditional medicines in Africa | Kasilo, OMJ; Wambebe, C; Nikiema, JB; Nabyonga-Orem, J | 2019 | BMJ GLOBAL HEALTH | Include | Survey data on contribution of traditional medicine to UHC in African countries |
