# Supplementary material for: Do private health providers help achieve Universal Health Coverage? A scoping review of the evidence from low-income countries
Source: Health Policy Plan. 2023 Aug 21;38(9):1050–63. doi: 10.1093/heapol/czad075 (PMC10566321; doi:10.1093/heapol/czad075)
Supplement: czad075_Supp [file czad075_supp.zip › Appendix 3_Final list of papers included in the review, with evidence extracted.docx]

## Appendix 3: Final list of papers included in the review, with evidence extracted

| **First author, Date, Journal** | **Country/ setting** | **Evidence type** | **Study purpose** | **Sample/ data sources, Private sector** | **Key findings** | **JBI score (%)** |
| --- | --- | --- | --- | --- | --- | --- |
| Abiiro et al., 2014  BMC Health Service Research | 6 rural communities in Malawi | Qualitative interview and focus groups | Understand perceived gaps in population coverage and financial protection in rural Malawi | Focus groups with 127 community residents  Interviews with 8 health workers (4 public, 2 PFP and 2 CHAM) Aug-Sep 2012  Formal PFP clinics  Formal PNFP CHAM | P: PPs were better distributed geographically for the pop. – public facilities were a long distance and patients reported arriving after opening hours. CHAM HCWs reported only being funded to provide maternal care under SLA, excluding paeds services.  F: Community members reported widespread OOPP for PFP/NFP, often requested before treatment.  Q: Community members reported higher quality in both PP types than public – better facilities and more time with HCWs. Patients reported better availability of drugs, HCWs and equipment at PPs. | 80 |
| Ameh et al., 2021  BMC Fam Pract | Seven communities in East and West Africa. Four Nigerian communities and one each in Kenya, Uganda and Tanzania | Qualitative interviews and focus group discussion | Describe perceptions of healthcare users, providers and other stakeholders on health-seeking, access to and quality of PHC | 155 healthcare users for 24 focus group discussions. 25 healthcare users, providers, and stakeholders for in-depth interviews. 11 providers and stakeholders for key informant interviews  Unspecified private facilities | Q: Community members reported quality being better in PPs compared to public sector, for example shorter wait times and staff having fewer patients. | 80 |
| Atake, 2020  BMC Health Serv Res | Togo, Lomé-Commune region | Quantitative cross sectional household survey | Understand whether type of health insurance affects health service utilisation, provider choice and financial protection of households in Togo | Household questionnaire / interview  1180 insured households  May 2016  Formal PFP insurance and health centres | F: Public and private policyholders were of similar age, household size and head of household gender.  OOPP was reported as the main barrier to accessing health centres among publicly insured households (49%) and the second reason for privately insured households (43%). Privately insured households were more likely to use private health providers than publicly insured.  Monthly OOPP per household was estimated at 31,657 CFA / 55USD for publicly insured and 24,000 CFA/42USD for private.  No significant difference in CHE between publicly and privately insured households, when CHE threshold varied from 5-40%: 62-4% publicly insured and 61-4% for private. | 80 |
| Benova et al., 2018  BMC Health Serv Res | Uganda | Secondary quantitative data | Understand provision of antenatal care coverage in Uganda | DHS data for 1995, 2000/01, 2006 and 2011  All non-state PFP/NFP combined | P: From the 2002-06 period to the 2007-11 period fewer women used private sector ANC - signif decreased from 1.5m (19.2%) to 0.9m (13.7%) compared to public 6.1m.  For deliveries, women using private facilities declined significantly from 42.1% (1995) to 25.1% (2011).  Between 2007–11, the public sector provided care for 3.2m deliveries and the private sector for 1.1m. An absolute increase for both, from 0.9m (public) and 0.6m (private) in 1991–1995.  Overall, women from the richest households, with higher education levels and living in urban areas were most likely to use the private sector for ANC and deliveries.  Q: No overall stat signif difference between public and private sector in the provision of all recommended components of ANC or delivery care. Of those who did receive all 8 components of recommended ANC, more women used the private sector for (17.5%) vs 8.5% in public sector. | 100 |
| Beogo et al., 2016  BMC Res Notes | Ouagadougou, Burkina Faso | Quantitative cross-sectional survey of households | Understand medical expenditure in an urban setting with a developing private sector | Questionnaire carried out  Aug -Nov 2011  1017 households  1573 individuals participated  Formal private facilities (PFP & PNFP).  Informal sources (self-treatment, traditional healers, and marabouts) | F: Treatment at public teaching hospitals was on average the most expensive, followed by private for-profit health facilities - mean expenditure at private NFP providers was about half of PFP. District hosp closely followed, then primary healthcare centre. Nurse-led PNFP was the cheapest among formal providers.  Informal care providers were 14% cheaper than the overall sample mean.  Self-medication was the cheapest option overall.  Patients using private providers paid 50% more for medicines than for public services and twice as much for consultations - this ranged widely depending on the type of service (PHC v specialist). Overall expenditure on private facilities was 48% more than in public facilities and 141% more than those who treated themselves. | 100 |
| Beogo et al., 2016  Acta Trop | Ouagadougou, Burkina Faso | Quantitative cross-sectional survey of households | Understand use and OOPP to public & private malaria treatment in Burkina | Questionnaire  Aug - Nov 2011  715 households  1082 individuals with self-reported malaria  Formal PFP  Formal NFP (NGO and faith-based)  Informal PFP  Informal NFP  (Self treatment) | P: Of 228 patients who had intravenous treatment, 142 in public facilities (77% in PHCs) and 86 in private clinics  P: Just over a quarter of respondents (27.4%) accessed care from private providers (14% from PFP (5% doctors and 9% nurses) and 12% from PNFP (9% doctors and 3% nurses). 2% were treated in private at home. The public sector was visited by >one-third (n = 415) of participants [primary health centre (PHC): 29.2%; district hospital: 8.3%, teaching hospital: 0.8%]. 34.2% (n = 370) self-medicated, and 0.1% (n = 1) used a traditional healer.  F: Costs were significantly higher (p <0.001) for patients using private providers compared to public - mean avg household expenditure per malaria episode In USD: $24.5 for private (both PFP and PNFP), $17 for public and $5.2 for informal provision.  Patients using PFP doctor-led facilities had the highest expenditure ($50.4 per treatment), followed by public teaching and district hospitals ($37.4 and $23.1), then PFP nurse-led ($19.8) closely followed by PNFP doctor-led ($19.6). | 100 |
| Campbell et al., 2016  Trop Med Int Health | 57 LMICs | Quantitative cross sectional survey at multiple time points | Assess the role of the private sector in providing reproductive and maternal/newborn health services across LMIC, and socio-economic groups | DHS data for 2000–2013  865,547 women aged 15–49  Whole sector - incl NGO and faith-based providers and non-medical vendors (shops, pharmacies, drug sellers, nightclubs) | P: Approx % women in need using modern family planning methods from private and public sector:  Rwanda 4% and 56%  Malawi 17% and 43%  Ethiopia 9% and 41%  Madagascar 10% and 32%  Burkina Faso 9% and 30%  Mozambique 5% and 27%  Uganda 25% and 22%  Liberia 10% and 12%  Niger 8% and 11%  Mali 7% and 10%  Chad 2% and 5%  DRC 9% and 4% | 100 |
| Chakraborty et al., 2018  Int J Equity Health | Bangladesh Cambodia DRC Dominican Republic Ghana  Haiti Kenya Liberia Mali Nigeria Senegal Zambia | Quantitative cross sectional survey | Understand use and source of family planning, and children's care, by wealth quintile | DHS data for:  DRC 2013/14: 18,827 women and 10,701 children  Liberia 2013: 9239 women and 5072 children  10,424  Mali 2012/13: women and 6439 children  Formal PFP/NFP Informal unspecified | S: Family planning In Mali 58% of FP services were provided by government community health workers, more in rural areas. About a fifth were provided by private pharmacies, about 5% private clinics and 5% other sources.  DRC had a large proportion of private pharmacy use, especially in urban areas.  About 20% of Liberia's services were private clinics, about 10% private pharmacies - about a quarter of urban services were private clinics and about 15% in rural places.  Use of private providers increased with wealth quintile in all countries.  Diarrhoea:  Overall, private clinics and pharmacies provided care for about 25% in DRC, 20% in Liberia and 10% in Mali.  Informal providers accounted for 42% in Mali, 10% in DRC and 20% in Liberia.  The proportion of private providers increased in urban areas and informal care increased in rural settings.  In Liberia, the wealthier are more likely to use private facilities.  Fever:  Around a quarter of DRC and Liberia fever cases were treated in the private sector, while only about 10% in Mali were.  About 25% were treated by the informal sector in Mali - 65% in rural areas, 15% in urban areas. However 54% of fevers were not treated at all.  35% treated in private pharmacies in urban DRC and 20% in private clinics.  Minimal or no NGO involvement for any of the conditions. | 80 |
| Chirwa et al., 2013  Health Research Policy And Systems | Rural Malawi | Mixed methods - survey, interviews, and  secondary data from health facility databases | Understand how contracting faith-based private providers contributed to expanding UHC in Malawi | 155 maternal and child health clients  5 health facilities  Formal faith-based PNFP | P: Unanimous agreement by participants that the SLA had reduced geographical barriers and increased population access to health care especially for the poor, and improved equity.  Service use increased significantly following SLA introduction: in one facility the number of deliveries increased 400% and paediatric visits increased 600%.  S: Selected essential health package (EHP) services for women and children under five covered by a service level agreement (SLA) were offered by the provider for free.  S: Unanimous agreement by participants that the SLA had increased coverage of health services  Q: Issues with future sustainability were raised as workload and costs mounted with increasing referrals - without increase in resources, threatening quality of care. | 70 |
| Doctor et al., 2019  J Glob Health | Sub-Saharan Africa | Quantitative cross-sectional survey for two time periods | Identify trends in the scale and inequalities of private & public facility use for childbirth in SSA countries | Secondary DHS data for 2000-07 and 2008-16  25 SSA countries incl 12 LICs (Burkina Faso, Chad, DRC, Ethiopia, Guinea, Liberia, Malawi, Mali, Mozambique, Niger, Rwanda, Uganda)  Formal PFP and PNFP combined (faith-based/NGO) | S: Between the two survey periods, Ethiopia began with the lowest overall rate of use of private facilities (0.3%), increasing to 1.4%.  The DRC began with the highest level of private facility births (20.5%) decreasing to 15% - the biggest decrease in use.  Private facility births:  Decreased by 58.7% in Rwanda  Increased by 435% in Ethiopia  P: Malawi had the highest utilisation of private facilities by poor women, at just over 10%.  The difference in private facility use by wealthy and poor quintiles was significant. | 100 |
| Ewen et al., 2017  PLoS One | 30 low-income and middle-income countries | Quantitative cross-sectional survey | Assess availability and affordability of NCD meds in private and public providers | Secondary analysis of WHO/HAI database ​​  2161 medicine outlets surveyed from 2008 - 2015  Unspecified PFP providers | S: Overall in LICs, 15.2% and 18.9% of lowest priced generics met WHO availability and affordability target in the public and private sectors, respectively - ranging from 11.9% (CVD) to 23.5% (COPD) in the public sector, and 11.4% (CNS) to 27.8% (diabetes) in the private sector. The proportion of all lowest-priced generics meeting the target increased with country wealth.  The main cause of unaffordability of cheapest generics and originator bands in private sector was low availability (48.9% and 68.5%, respectively) | 67 |
| Gele et al., 2017  Risk Manag Healthc Policy | Mogadishu, Somalia | Qualitative | Explore the accessibility and people’s trust in the private sector Somalia | Unstructured interviews 7 doctors who own private health facilities, 8 patients, 5 medical students and 3 MOH officials  Aug-Nov 2016  Formal PFP – health facilities and solo practitioners | P: Participants agreed that healthcare is predominantly provided by the private sector. Some considered it to be filling a gap left by the public sector. Most considered that no one would access care at all without the private sector. One person claimed that PPs only open when the owner wants to.  F: Private health centres owned by doctors charged an avg fee of $8 ($3-$10).  Patients reported private providers being unaffordable to many and prescribing unnecessary treatments in order to increase profits.  Q: Participants agreed that PPs are free to do what they wish without any regulatory framework to ensure standards of quality or efficiency. Many participants agreed that providers were more concerned with profit than quality of patient care. Inappropriate prescription was regularly mentioned as a problem, driven by providers wishing to use up stock. Some also reported feeling disrespected by PPs asking for payment before accepting them as patients, and being treated in a transactional way. | 90 |
| Grépin, K.A. 2016  Health Affairs (Millwood) | LMICs | Quantitative cross-sectional survey | Provide estimated levels and trends in the use of private maternal, child, and reproductive health care services in low- and middle-income countries | DHS for 1990–2013  230,000– 350,000 sick children, 1.6 million recent births (and on antenatal care for roughly one-third of those births), and 730,000 women using modern contraception  Whole sector included formal PFP and PNFP and informal PFP | P: Approx private sector use for LICs v LMICs v UMICs:  Diarrhoea 52% v 74% v 32%  Fever/cough 55% v 76% v 34%  Institutional delivery 22% v 47% v 12%  Antenatal care 25% v 48% v 34%  Modern contraception 36% v 38% v 42% | 100 |
| Hanson et al., 2017  Malar J | 8 SSA malaria-endemic countries: Benin, DRC, Nigeria, Kenya, Tanzania, Uganda, Madagascar and Zambia | Quantitative cross-sectional survey | Identify trends in availability, price, and market share of malaria diagnostics in the public and private sector | DHS data for 1990–2013  198,836 outlets  Formal PFP hospitals & clinics  Informal PFP drug vendors & unlicensed stores | S: The private sector price of malaria rapid diagnostic testing (RDT) for children was the same as quality-assured artemisinin combination therapy (QAACT) in Madagascar but X 2.5 in DRC Katanga X 2.1 in Uganda.  Malaria microscopy was 3.3 X in DRC Katanga, 2.1 times more in Uganda and 8.3 times in Madagascar.  S: Availability of any type of malaria testing by PFPs increased significantly from 2009/11 to 2014/15 in Kinshasa DRC (82.1 to 94.0%), Uganda (47.1 to 70.1%) and Madagascar (14.5 to 45.0%) but decreased in Katanga DRC (72.7 to 55.6%).  Most malaria testing was done in the public sector using RDTin 2015: Madagascar (84.5%), Uganda (71.8%), and the DRC Katanga (74.3%) and Kinshasa (51.9%). | 100 |
| Kasilo et al., 2019  BMJ Global Health | 47 countries in SSA | Qualitative interview | Understand how R&D in African traditional medicine can impact practice and UHC | Questionnaire  Ministry of Health officials from 47 countries  Informal PFP: traditional medicine | S: Burkina Faso’s Centre for Experimental Research evaluates traditional medicines (TM) used by traditional health practitioners (THPs) and has found new safe and effective treatments for sickle cell disease management in children and for opportunistic AIDS-related infections. Uganda has patented at least five TMs, including for malaria and benign prostate hypertrophy.  Madagascar has a patent for a TM to treat type II diabetes.  Mali screened 66 plants used by THPs and found a safe and effective malaria treatment that was a lot cheaper than existing treatment.  S: Functioning collaborations between THP and certified health practitioners (CHPs) that integrate THPs into the primary health delivery system reported for: Burkina Faso, CAR, Ethiopia, Malawi, Mali, Mozambique, Niger and Uganda.  Q: Institutionalised training programmes for THPs reported for: Burkina, Mali, Mozambique, Niger and Uganda.  Drug registration systems that include TM products were reported for: Burkina, CAR, DRC, Ethiopia, Madagascar, Mali, Mozambique, Niger, Togo and Uganda.  Mali has a dedicated research centre to evaluate TMs. The Dept of Traditional Medicine facilitates education and joint capacity building workshops for THPs and CHPs and registers THPs who meet set competencies. This is supported by 51 THP associations and a THP federation. Only certified THPs are allowed to market their products. | 33 |
| Khuluza et al., 2017  PLoS One | Southern Malawi | Quantitative survey | Analyse availability and affordability of malarial medicine provided by private & public sectors in Malawi | Survey  31 health facilities and medicine outlets (10 public health centres, 4 public district hospitals, one public central hospital, 8 CHAM facilities, 2 licensed pharmacies, 3 licensed drug stores and 3 non-licensed street vendors)  6 antibiotics and 6 antimalarials  Formal PFP (pharmacies, drug stores)  Formal PNFP (CHAM)  Informal PFP (unlicensed street vendors) | F: Public facilities provided drugs for free. A small number of medicines were provided free from some CHAM facilities.  PFP prices were lower than CHAM with respect to international reference prices  S: 68% of public sector meds were generics, 58% in CHAM and 13% of the PFP sector.  Availability for 4 core drugs: central public hospitals (100%), district hosps (100%), CHAM (78%), private pharmacies (88%), public health centres (65%), licensed drug stores (42%) and illegal vendors (33%).  For all 12 medicines: 67% availability in central public hosps, 58% in district hosps, 27% in public health centres - 52% in CHAM - 92% in private pharmacies, 28% drug stores and 22% illegal vendors.  Q: No samples from CHAM facilities failed quality tests. 1 from public facilities did. 4 samples failed quality testing from formal PFP and 2 from informal PFP. | 100 |
| Kiguli-Malwadde et al., 2020  Pan Afr Med J | Uganda | Quantitative cross sectional | Audit the distribution of radiology equipment in Uganda | Survey  354 public and private health centres  625 pieces of imaging equipment | P: Most of the equipment was located in the central region where the capital city is, despite the population being fairly evenly distributed across Uganda's four regions.  P: The private sector owned:  53% of 397 pieces of 'plain radiography'  62.5% (20/32) of fluoroscopy devices  17/20 85% of mammography equipment  All 3 radiotherapy devices  18/25 CT scanners  89/120 dental radiography | 67 |
| Kim et al., 2016  BMC Public Health | Afghanistan | Quantitative cross sectional | Explore equality and equity in use of inpatient/OP/maternal health services in public and private facilities | Survey  15,688 households with 18,255 pregnant women  Formal PFP (hospitals and clinics | P: Women in urban areas had higher overall rates of ANC and delivery service use than rural women. They were more likely to use PFPs for ANC services (55.6%) but deliver in a public facility (51.2%) with only 14% private delivery. Women from rural areas mainly used public facilities for both, only 21% had private ANC and 3% private delivery.  There was a wealth gradient in use of PFPs. 51% of the wealthiest used private ANC and 15% for delivery. 11% of the poorest used private ANC and <1% private delivery.  P: Households used private facilities more for outpatient care (12.7% vs. 6.7%; p < 0.001) and public facilities more for inpatient care (3.3% vs. 0.7%; p <0.001) | 63 |
| Montagu et al., 2021  Frontiers in Med (Lausanne) | low- and middle-income countries | Quantitative analysis of DHS data | Identify the source of inpatient and outpatient care across LMICs | Demographic and Health Surveys  65 LMICs  Unspecified private sector | S: Uganda was the most privatised LIC and country: 40.2% of all care privately delivered (21.4% of inpatient and 56.9% of outpatient care) | 100 |
| Nabyonga Orem et al., 2011  Health Policy Plan | Uganda | Quantitative analysis of cross-sectional survey data | Understand the effect of user fee abolition on progressing UHC in Uganda | Uganda National Household Survey datasets for 1999/2000, 2002/03 and 2005/06  Between 10696 households with 57385 individuals in 1999/20 and 7426 households with 39322 individuals in 2005/06  Formal PFP hospitals, clinics, health centres, drug shops, pharmacies  Informal PFP: traditional doctors | P: Overall, the private sector was consistently the most used source of healthcare.  Private hospital use decreased year on year: 52% from 1999/00 - 2002/03 and 37% to 2005/06.  Private clinics were the main source of care for both richest and poorest in 1999/00 - about 32% and 47%, respectively - in 2005/06 after fees were abolished, the poor mostly used Gov health centres (41%) and private clinic use by the rich increased by 20% (providing approx 68% of first consultation)  THs provided around 3% of first consultations for the poor and >1% for the rich in 1999/00 - and saw a signif decline in use by 2005/06.  Private pharmacies and drug outlets were the first place of consultation for around 12-16% of people, without significant difference between quintiles or change over time.  F: Households annually spent on avg 62USD on traditional healers in 1999/00 (before public sector user fee abolition) - more than any other category of care for both richest and poorest quintiles. Hospital/clinic charges (for public and private care combined) became the highest area of spending in 2005/06 (after public fees were abolished) at 116USD and TM rose to 71USD. | 80 |
| Namakula et al., 2021  Glob Health Action | Northern Uganda, Gulu district | Mixed methods | Understand prevalence and mechanisms used by for-profit health providers to expand access to poor populations in Uganda | Survey and interviews  45 PFP providers  Formal PFP (clinics, laboratories, pharmacies, medical centres, hospitals, insurance provider clinics, drug shops, medical imaging centres)  Formal PNFP: NGO clinics | P: Some of the better established FPFPs reported providing free immunisation, supported by Gov subsidies in the form of vaccines, fridges and PHC thereby contributing to the district's pop coverage. Some also reported partnerships with NGOs to provide free testing and family planning. FPFPs advertised their other paid services when providing free services.  F: 13/45 PPs reported offering fee exemptions (6 PFP clinics, 3 med centres, 2 drug shops and 1 NGO clinic - but none of the pharmacies, labs, hospitals or insurance clinics).  Categories exempted included: elderly, adolescents, pregnant women, child mothers and disabled. Mostly applied to basic services: consultation, prescription and sale of medicines, HIV and Hep B testing, and family planning.  Some exempted fees for immunisation of children, diabetes screening and blood transfusion.  More complex services such as X-ray, surgery and child delivery were less likely to be included.  The same pattern was seen for price reductions, but this was extended to regular customers, the uninsured and 'those who can't afford it'.  FPFPs used 'price discrimination' - varying fees across percieved wealth of clients to make up for losses - and bargaining with clients. They tried to keep this practice private to prevent wealthier clients asking for discounts.  Loans books were used by smaller providers to record deferred payment, or instalments to reduce financial burden. Breaking down doses - paying for the used bit and returning to receive/pay for the rest the next day - esp for malaria.  Q: Concerns about quality were reported with respect to breaking down doses and clients not returning to receive full treatment. | 80 |
| Ntambue et al., 2018  PLoS ONE | Maternity wards in Lubumbashi, the DRC | Quantitative cross-sectional | To understand variability in user fees for maternal care in public and private facilities | Survey  1,390 women  1-8 March 2014  Formal PFP (health facilities)  Formal PNFP (health facilities) | F: Women in public facilities paid less than private sector for delivery but more for caesarean. VD was cheaper in PNFP compared to private polyclinics.  No significant difference in cost for caesareans between public and PNFP compared to PFP. PFP charged US$63 for emergency care and public $52  Vaginal delivery was more cost effective in the PFP and faith-based sector than public or parastatal. ICER = 56.  P: 471 deliveries were carried out in public facilities, 100 in parastatal, 685 in PFP and 134 in PNFP. | 100 |
| Nyasulu et al., 2019  Glob Pediatr Health | Malawi | Quantitative cross-sectional survey | Understand sources of diarrhoea treatment for under 5’s in Malawi and associated factors | DHS for 2015-16  3584 children/their guardians  Formal PFP/PNFP (hospitals, clinics, doctors, mobile clinics, pharmacies, shops, Malawi Aids Counselling and Research Organization)  Informal drug outlets, youth centre  Unspecified | P: Of 3584 children, 2393 (66.8%) sought treatment or advice for their diarrheal episode - of which 80% went to a public facility, 9% to a private provider, 8% to retail drug outlets, and 3% to unspecified.  Children in the highest wealth quintile (20.7%) sought care from private health facilities 4 times more than those in the lowest (5.7%) and second lowest quintiles (5.2%). Children in the richest quintile were more likely to use private facilities and drug outlets than public facilities.  16% of urban residents used private facilities compared to 7.5% of rural residents. However rural residents were more likely to seek care from private than public health facilities.  The likelihood of choosing a retail drug outlet for treatment over a public facility increased with the child's age | 100 |
| Orya et al., 2017  BMC Health Serv Res | Bombali district in Sierra Leone and Maroodi Jeex region of Somaliland | Qualitative | Understand the role of traditional birth attendants in increasing access to maternal health services in fragile settings | Interviews and focus groups  49 service users, 39 TBAs, 7 HCWs, 4 managers, 3 health programme staff, 2 village chiefs.  Formal/Informal PFP - TBAs | P: A key theme in interview feedback was that trained TBAs acted as a link to healthcare, increasing uptake of formal health care at facilities by pregnant women, by advising them and explaining the risks and benefits, taking them to health centres and promoting institutional delivery.  In Somaliland, document analysis showed that referrals increased to 56% of deliveries in the 6mo following TBA training, 72% were referred in year 2 and 67% in the third year. The number of women receiving care from maternity services increased from 779 in 2009 to 3296 in 2012. Facility deliveries also increased.  Participants in both settings reported improved relations between health centres and TBAs and strengthened integration of the services/providers, and the important role of both in increasing access to care. TBAs provide community outreach while health centres provide safe health care.  Trained TBAs helped patients to overcome geographical, distance and transport barriers by accompanying them.  Q: In interviews and discussions: before TBAs received training they were perceived to provide low quality service that did not improve health outcomes. Following training, participants perceived the quality to have increased, with TBAs more skilled and knowledgeable about how to spot danger signs and refer/increase access and use of health facilities and adherence to medication instructions. | 80 |
| Palafox et al., 2019  SSM Population Health | Benin, Nigeria, Uganda and Zambia | Quantitative cross-sectional | To understand physical and financial access to malaria treatment by sector and location | Household surveys  Uganda:  1,300 households, 1,300 outlets (2009-10) and 1,500 households, 3,300 outlets (2011-12)  Formal PFP (health facilities, pharmacies, drug stores)  Informal PFP (market stalls and kiosks) | P: In Uganda urban households had >90% coverage rising to complete coverage in 2011/12.  About 70% rural households had access in 2009/10 rising to >90%.  The urban-rural ACT access gap was mostly eliminated by 2012: >95% of households in both areas had access to at least one source of ACT within 5 km.  F: Median cost of ACT sold by PFP was $5.85 in urban settings decreasing to $3.10 and in rural settings $3.86 to $1.86. | 100 |
| Pettigrew et al., 2016  Int J Equity Health | LMICs - including Madagascar, Rwanda, Burkina, DRC, Malawi, Togo and Niger | Quantitative | Identify PHI expenditure trends and drivers in LMICs | WHO's Global Health Expenditure Database  188 LMIC health accounts teams 1995–2012  Formal PFP - insurance providers | F: Between 1995 - 2012 OOPP was:  <20% of THE in Malawi  20-40% of THE in Madagascar, Rwanda, Burkina and DRC  >40% in Togo and Niger  During the period VHI rose and OOPP fell in Madagascar, Rwanda, Burkina and DRC.  VHI and OOPP fell in Malawi, Togo and Niger. | 80 |
| Poyer et al., 2015  Trop Med Int Health | 9 LMICs including 2 LICs: Madagascar and Uganda | Quantitative cross-sectional | Identify the public/private market share of malaria rapid diagnostic tests in LMICs | National surveys:  773 public/NFP outlets and 509 PFP outlets in Madagascar  830 public/NFP outlets and 2373 PFP outlets in Uganda  Formal PFP - health facilities, pharmacies and drug shops | F: RDTs were free in public health facilities, NFP and from CHWs in both countries  PFP in Madagascar offered free RDTs while pharmacies charged $3.67 and $0-$0.15 in drug shops  In Uganda PFPs charged on avg $1.20 and pharmacies $0.88 and drug shops $0.80  S: Availability of any RDT was much higher in public and NFP outlets than PFP in Uganda and even more so in Madagascar where it was less than 10% in any type of outlet, while >90% in public health facilities.  The number of RDTs sold/given out was generally higher for public health facilities than private:  Uganda - median 24 public v 5 in PFP centres  Madagascar - median 5 in public health facilities v 4 in PFPs.  S: The largest range of RDT brands available was in Uganda, 9 in public/NFP sector and 13 additional brands in PFP. | 83 |
| Ravindran et al., 2011  Reprod Health Matters | 27 countries across Africa, Asia and Latin America | Mixed methods | Understand if social franchising contributes to universal access to reproductive services | Document review:  Documents for  45 social franchises from a compendium published by the University of California  Formal PNFP (social franchises) | S: DRC: 1 franchise started 2003, clinics and pharmacies providing limited services, see 14 patients per day per outlet  Ethiopia: 1 franchise, start 2007, clinics, limited services, see 3 patients per day per outlet  Madagascar: 3 franchises started 2001-10, clinics, provide limited services, see 1-3 patients per day per outlet  Malawi: 1 franchise, started 2005 clinics, limited service, see 1 patients per day per outlet  Togo: 1 franchise, started 2009, clinics and hospitals, provide full range of services, see 0.32 patients per day per outlet  Uganda: 2 franchises, started 2007/08, fractional clinics see avg 0 patients per day per outlet, full service CHWs see 2 patients per day per outlet | 60 |
| Riley et al., 2018  PLoS One | Ethiopia, Nigeria, and DRC | Quantitative cross-sectional | Understand private sector market share and availability/affordability of contraceptives in Ethiopia, Nigeria, and DRC | Survey  8,295 outlets in Ethiopia and 2,207 in DRC  2015  Formal PFP (clinics, pharmacies, drug shops and general retailers) | F: In DRC, prices per oral contraceptive and ECP dose were 25–166% higher in pharmacies than in drug shops. Prices per-dose for injectables were highest in private clinics across all countries, potentially due to the inclusion of an administration service fee.  Median price per unit in USD:  Oral Contraceptives  Ethiopia: private clinic ($0.15) Pharmacies ($0.36) Drug shops ($0.15)  DRC: private clinic ($0.33) Pharmacies ($0.88) Drug shops ($0.33)  Emergency Contraceptive Pill (ECP)  Ethiopia: private clinic ($0.49) Pharmacies ($0.49) Drug shops ($0.49)  DRC: private clinic ($0.55) Pharmacies ($1.65) Drug shops ($1.32)  Injectables  Ethiopia: private clinic ($0.34) Pharmacies ($0.24) Drug shops ($0.24)  DRC: private clinic ($1.65) Pharmacies ($0.55) Drug shops ($0.55)  Implants  Ethiopia: private clinic ($1.46) Pharmacies ($0.83) Drug shops ($0.97)  DRC: private clinic ($7.15) Pharmacies (-) Drug shops ($7.70)  IUDs  Ethiopia: private clinic ($1.46) Pharmacies ($0.24) Drug shops (-)  DRC: private clinic ($4.95) Pharmacies ($66.22) Drug shops ($2.20)  S: FPFP dominated the market for modern contraception:  Ethiopia - 85% of 8,295 outlets (including general retailers 74% of the market but only sold condoms). After excluding general retailers, 96% of outlets had available stock on the day of survey. Private clinics were the largest group of PFP outlets (25%).  DRC - 80% of 2,207 outlets (including drug shops 60% of market, and excluding general retailers). 41% had available contraception on the day of the survey.  Availability of modern contraception other than condoms was similar across private and public outlets:  Ethiopia (98% and 95% respectively)  DRC (47% and 42%).  Among PFP, availability was highest in pharmacies in Ethiopia (100%) and DRC (64%). Private clinics were least likely to have modern contraceptives available.  Only PFP clinics and public facilities were allowed to administer permanent and long acting methods, so short-acting methods were more available.  Higher availability in PFP than public sector:  Ethiopia - 56%-97% availability across methods - except emergency pill in private clinics (30%).  DRC < 50% availability of all methods across PFP.  PFP stock-outs seen in all countries (1–21%).  Availability of oral contraceptives and ECP highest in pharmacies (Ethiopia: 97% and 68%,DRC: just <50% and 44%, respectively).  LARCs (implants/IUDs) had low availability in PFP and only in clinics, not pharmacies or drug shops. Availability was higher in public sector:  Ethiopia (17–74%)  DRC (13–20%)  Availability of 3+ contraceptive methods excl condoms) was overall lower in PFP than public sector in both Ethiopia and DRC.  PFP availability of 3+ methods in DRC was <8%. | 100 |
| Salim et al., 2018  SAGE Open Med | Sudan | Qualitative | Explore health insurance providers’ perceptions of PHI contributions to UHC/financial protection in Sudan | Interviews  5 PHI providers and the 4 SHI providers  Formal PFP - insurance companies | P: All insurers admitted to offering insufficient levels of coverage - particularly PHI. PHI provides coverage for less than 0.5M people and reported socioeconomic disparities due to high premiums. National SHI covered 16M (51%) of individuals nationally and Khartoum State insurance covered 0.96M (72%) of State households.  F: PHI enrolment fees were much higher than SHI: 1500 - 6000 Sudanese pounds per person annually for PHI, compared to 40 SDG per household per month for informal sector workers or 10% of formal worker salary with 4% paid by the employee for SHI. All insurers provide care free at the point of delivery, except for medicines which patients co-pay 25% in SHI and up to 10% for PHI or less/nothing for higher premiums.  SHI didn't have financial ceilings for any care while PHI did for each package and specific service.  S: PHI providers offered a range of packages with different premiums or included services. SHI tended to have one package for all. All providers claimed their packages to be comprehensive. SHI operated a gatekeeping mechanism for specialist services via GPs, while PHI policyholders could go straight to specialists. Both SHI and PHI provided principal coverage of basic essential health services - consultations, investigations, hospital admission and surgery, rather than PHI just providing supplementary cover as in HICs. Some PHI covers treatment abroad but usually for higher premiums. PHI tended to purchase services from providers while SHI tended to also provide the services themselves/have greater involvement in service delivery.  SHI prescribed generics by default while PHI provided branded.  Q: PHI providers claimed to offer good quality services, as did SHI. Two commented on private hospitals offering higher quality service which PHI provides access to. PHI providers claimed to have convenient processes and large networks of providers. | 80 |
| Sisay et al., 2021  J Pharm Policy Pract | Eastern Ethiopia | Quantitative sectional survey | Identify availability and affordability of essential medicines in Ethiopia in private vs public sector | Questionnaire  30 public and 30 private health facilities  Essential medicines on WHO/HAI guidelines and Ethiopia’s essential medicine list  1-31 March 2020  Unspecified PFP | F: 94% (n=47) of LPGs were more expensive from PFPs than the public sector.  64% (n=32) had a significant price difference between (p<0.05).  Median prices were >3 time the reference price for 8 LPG versions of essential drugs  Most of the medicines from public and private outlets were unaffordable - cost >1 day wage. 79% overall - 72% for public and 92% private.  Top unaffordable products: ceftazidime, risperidone, and ampicillin injection - required 171, 73 and 56 days wage.  Private outlets: ceftazidime, risperidone and valproate cost 186, 95 and 63 days wages.  Public: risperidone, ceftazidime and valproate cost 71 days wage, 54 and 40.  Evidence that drugs are more expensive and less affordable in the private sector. Public sector also not affordable.  S: Overall availability of OB products in public sector was 1.43%  Private sector availability of OBs of 50 essential medicines was 5.5% (pharmacies 6% and and drug stores 4.5%)  Overall LPG availability was 50% (1.7% for bisoprolol and amiodarone to 93.3% for amoxicillin) including:  Public: 42.5%  Private: 50.8% (pharmacy=55.7% and drug store=42.83%)  Quant data showing that availability of essental drugs - both branded and generics - is higher in private providers | 100 |
| Ssennyonjo et al., 2018  Int J Equity Health | Uganda, the Uganda Catholic Medical Bureau (UCMB) | Mixed methods/case study | To understand government resource contributions (GRCs) to PNFP providers in Uganda | Document analysis: 36 docs related to Gov-PNFP relations and grants  Interviews: 39 key informants from Gov, PNFP and development partners.  Secondary quant data: UCMB database service delivery outputs between 1997-2015  Formal PNFP (Uganda Catholic Medical Bureau network) | F: Document review and interview data showed that services were offered at no or little charge while Government GRCs were increasing, but user fees increased as government funding decreased  S: The quant data show provision of OP services and deliveries gradually increased during study period as GRCs increased | 100 |
| Tan et al., 2021  BMC Complement Med Ther | Rwanda | Qualitative | Understand the role of traditional medicine providers in expanding access to care in Rwanda | Interview and participant observation  6 traditional healers and 15 community members  June-July 2019  Informal PFP (traditional medicine) | P: Participants reported easier access to traditional healers who lived near them and/or were part of their social network/community. Traditional healers were especially convenient for treating minor illnesses.  F: Some participants reported flexible payment methods to traditional healers who let them defer payment or exempted some patients due to social ties.  S: Participants reported that traditional healers were most effective at treating uburozi (poisoning) and health centres often referred patients to them for this service.  Q: Community members reported that traditional healers/medicine responded more effectively to their social, health and financial needs than the hospital or clinics. They also considered that they responded to culture-specific illness 'uburozi' (poisoning) more effectively than health centres. | 80 |
| Wang et al., 2011  Social Science & Medicine | 12 LMICs including Chad, Ethiopia, Guinea, Rwanda and Uganda | Quantitative | Understand the role of the private sector in providing HIV-related services | Analysis of cross-sectional DHS data  Chad 2004, Ethiopia 2005, Guinea 2005, Rwanda 2005, Uganda 2006  Formal PFP (hospitals, clinics, pharmacies | P: HIV testing overall ranged between 2-29% for Chad, Ethiopia, Guinea, Rwanda and Uganda.  24% Ethiopian women and Chadian men receiving HIV test from PFP  In Uganda around 17% of HIV services were privately provided and 16% NGO use among men and 7% among women.  Ethiopia had low levels of NGO use for HIV testing.  Chad and Guinea reported no NGO use.  STI care  Chad, Ethiopia, Rwanda, and Guinea had insufficient data to disaggregate private provision. In Uganda, PFPs provided 45% STI care for women and 58% for men.  Use of private HIV testing was highest among women in the highest quintiles. About 15% of the poorest women in Uganda used private HIV testing.  In Ethiopia, 99% women and 93% men using PFP were in the richest quintile.  Multivariate analysis found no association between wealth and private sector use in Rwanda or Uganda for HIV testing. In Uganda wealthier women were more likely to use private STI services. |  |

ACT: artemisinin combination therapy; CFA: Central African Franc; CHAM: Christian Health Association of Malawi; CHP: certified health practitioner; CNS: central nervous system; COPD: chronic obstructive pulmonary disease; CVD: cardiovascular disease; DHS: Demographic & Health Survey; ECP: emergency contraceptive pill; EHP: essential health package; F: Financial coverage; FP: family planning; FPFP: formal private for-profit; GRC: government resource contributions; HAI: Health Action International; HCW: health care worker; IUD: intrauterine device; LIC: low-income country; LMIC: lower-middle income country; LPG: lowest price generic; OB: originator brand; OOPP: out-of-pocket payment; P: Population coverage; PFP: private for-profit; PNFP: private not-for-profit; PHI: private health insurance; PPs: private providers; Q: Quality; RDT: rapid diagnostic test; S: Service coverage; SHI: Social health insurance; SLA: service level agreement; TBA: traditional birth attendant; THP: traditional health practitioner; TM: traditional medicine; UMIC: upper-middle-income country; USD: US dollar; VD: vaginal delivery.
